# LIF-dependent survival of embryonic stem cells is regulated by a novel palmitoylated Gab1 signalling protein

**DOI:** 10.1242/jcs.222257

**Published:** 2018-09-20

**Authors:** Linda Sutherland, Madeleine Ruhe, Daniela Gattegno-Ho, Karanjit Mann, Jennifer Greaves, Magdalena Koscielniak, Stephen Meek, Zen Lu, Martin Waterfall, Ryan Taylor, Anestis Tsakiridis, Helen Brown, Sutherland K. Maciver, Anagha Joshi, Michael Clinton, Luke H. Chamberlain, Austin Smith, Tom Burdon

**Affiliations:** 1 Division of Developmental Biology, The Roslin Institute and R(D)VS, University of Edinburgh, Midlothian, EH25 9RG, UK; 2 Strathclyde Institute of Pharmacy and Biomedical Sciences, University of Strathclyde, Glasgow G4 0RE, UK; 3 Division of Genetics and Genomics, The Roslin Institute and R(D)SVS, University of Edinburgh, Midlothian, EH25 9RG, UK; 4 Department of Biomedical Science, The University of Sheffield, Alfred Denny Building, Western Bank, Sheffield S10 2TN, UK; 5 Centre for Discovery Brain Sciences, University of Edinburgh, Edinburgh EH8 9XD, UK; 6 Wellcome Trust-Medical Research Council Stem Cell Institute, University of Cambridge, Cambridge CB2 1QT, UK

**Keywords:** Leukaemia inhibitory factor, Gab1, Embryonic stem cells, Palmitoylation, Stem cell survival

## Abstract

The cytokine leukaemia inhibitory factor (LIF) promotes self-renewal of mouse embryonic stem cells (ESCs) through activation of the transcription factor Stat3. However, the contribution of other ancillary pathways stimulated by LIF in ESCs, such as the MAPK and PI3K pathways, is less well understood. We show here that naive-type mouse ESCs express high levels of a novel effector of the MAPK and PI3K pathways. This effector is an isoform of the Gab1 (Grb2-associated binder protein 1) adaptor protein that lacks the N-terminal pleckstrin homology (PH) membrane-binding domain. Although not essential for rapid unrestricted growth of ESCs under optimal conditions, the novel Gab1 variant (Gab1β) is required for LIF-mediated cell survival under conditions of limited nutrient availability. This enhanced survival is absolutely dependent upon a latent palmitoylation site that targets Gab1β directly to ESC membranes. These results show that constitutive association of Gab1 with membranes through a novel mechanism promotes LIF-dependent survival of murine ESCs in nutrient-poor conditions.

## Abstract (precis)

**Highlighted Article:** Embryonic stem cells rely upon expression of a novel lipid-modified Gab1 adaptor protein to promote survival under nutrient-poor conditions.

## INTRODUCTION

Embryonic stem cell (ESCs) are pluripotent cell lines derived from the inner cell mass of the blastocyst. They are immortal, differentiate into all foetal cell types *in vitro*, and most remarkably, when reintroduced into an appropriately staged embryo, reinitiate normal development to form all foetal tissues, including the germ line (Martello and Smith, 2014). These characteristics make ESCs a powerful experimental system and underpin their potential as a biomedical resource that can provide unlimited, scalable sources of normal cell types for regenerative therapy and drug screening.

The biological capacity and value of ESCs relies upon the fidelity with which their developmental potential can be maintained in culture and as a consequence, considerable efforts have been made to understand the role of self-renewal signals and the transcriptional factors that maintain ESC pluripotency (Ying and Smith, 2017). The cytokine leukaemia inhibitory factor (LIF) has a crucial role in promoting self-renewal of mouse ESCs, and the signals it elicits have also been shown to have an important physiological role in supporting preimplantation mouse development (Nichols et al., 2001). Although the requirement for this cytokine in ESCs of other species is unclear, LIF is included as a supplement in culture media that support human stem cell lines thought to be equivalent to mouse ESCs, and non-rodent pluripotent stem cells can respond to LIF, suggesting that this cytokine signalling pathway may have a general role in supporting pluripotent stem cells of mammals (Guo et al., 2016; Takashima et al., 2014; Theunissen et al., 2014; Thomson et al., 2012).

LIF-dependent signalling is initiated by heterodimerisation of LIF receptor (LIFR) with gp130 (also known as IL6ST), leading to cross-phosphorylation of receptor associated JAK tyrosine kinases. The activated kinases then phosphorylate specific receptor tyrosines that in turn serve as docking sites for the recruitment and activation of downstream effectors. The key effectors include the transcription factor Stat3, which activates expression of target genes directly, and the tyrosine protein phosphatase SHP2 (also known as PTPN11), which triggers the Erk/MAPK cascade and phosphoinositide 3-kinase (PI3K) signalling. Although studies have shown that Stat3 activation of the target genes Tfcp2L1, KLF4 and Gbx2 are critical for effective ESC self-renewal (Ying and Smith, 2017), the contribution of LIF activation of Erk/MAPK and PI3K pathways in ESCs is less clear (Niwa et al., 2009). Initial studies showed that SHP2 activation is not essential for self-renewal and that general suppression of Erk/MAPK signalling restricts mESC differentiation (Burdon et al., 1999). Indeed, suppression of Erk (ERK1/2, also known as MAPK3 and MAPK2, respectively) signalling when combined with activation of Wnt/β-catenin signalling very effectively promotes self-renewal of undifferentiated ESCs (Buehr et al., 2008; Li et al., 2008; Wray et al., 2011; Ying et al., 2008). This two-inhibitior (2i) culture system is enhanced further by the addition of LIF, suggesting that Stat3 or other LIF-induced signals are required to support efficient and robust propagation of ESCs (Dunn et al., 2014; Li et al., 2008; Ying et al., 2008).

A candidate mediator of LIFR signalling is Gab1 (Grb2-associated binder protein 1), an insulin receptor substrate (IRS)/Daughter of Sevenless (DOS) family adaptor protein (Holgado-Madruga et al., 1996; Weidner et al., 1996). In common with other IRS/DOS proteins, Gab1 has no intrinsic enzymatic activity but functions as a molecular scaffold to recruit and assemble signalling complexes. Gab1 is an important mediator of Erk, PI3K and PLC signalling induced by many growth factors, including LIF- and IL-6-related cytokines (Nishida and Hirano, 2003; Takahashi-Tezuka et al., 1998). The key functional elements of Gab1 include a membrane-binding activity encoded by the N-terminal pleckstrin homology (PH) domain, and an extended unstructured C-terminal region that mediates protein-protein interactions with other signalling molecules. The 110 amino acid PH domain contains seven β-sheets capped off with a short amphipathic α-helix, forming a classic PH-fold that contains a binding site for the phospholipid product of PI3K, phosphatidylinositol (3,4,5)-trisphosphate (PIP3) (Maroun et al., 1999a). Although initial recruitment of Gab1 to activated receptors is mediated through protein-protein interactions, tyrosine phosphorylation of Gab1 by receptor-activated kinases generates docking sites for PI3K, which, in turn, produces the PIP3 phospholipid ligand for the Gab1 PH domain. This PI3K domain- and PH domain-dependent positive feedback loop stabilises the interaction between Gab1 and activated receptors and amplifies downstream signals such as Erk via recruitment of SHP2 (Rodrigues et al., 2000). Previously, we have shown that Gab1 is phosphorylated by activation of the gp130 receptor in mouse ESCs (Burdon et al., 1999). Here, we report that the predominant form of Gab1 in mESCs is an unusual variant that lacks most of the conserved PH domain, including the PIP3 phospholipid binding site. Nevertheless, we unexpectedly find that this novel form of Gab1 is constitutively associated with ESC membranes and specifically promotes LIF-mediated ESC survival under conditions where nutrient availability is limited.

## RESULTS

### A novel short form of the Gab1 adaptor protein is highly expressed in ESCs

**Fig. 1.**
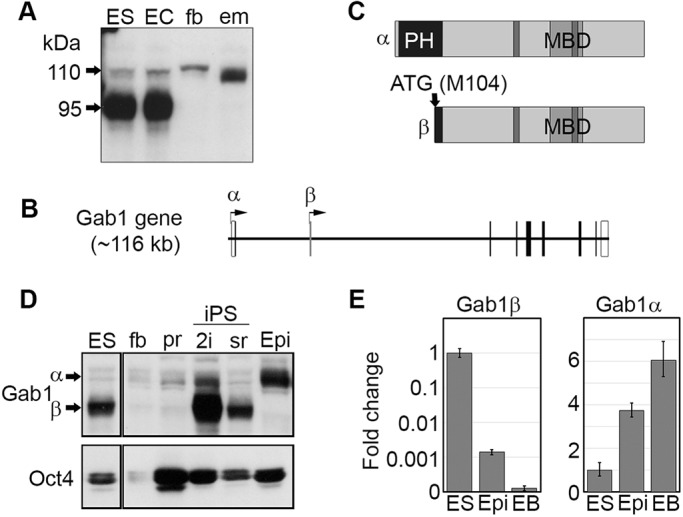
**Expression profile of a novel Gab1 variant protein.** (A) Gab1 western blot analysis of whole cell lysates from ESCs (ES), embryonal carcinoma (EC), 10T1/2 embryonic fibroblasts (fb) and pooled E12.5 embryo tissues (em). The positions of the 110 and 95 kDa Gab1 proteins are indicated. (B) Schematic of the *Gab1* gene locus, showing the location of the transcription start sites for Gab1α and Gab1β, and the exons. (C) Schematic showing the structure of Gab1α and Gab1β proteins. Dark grey and light grey areas are proline rich-regions and the Met binding domain (MBD), respectively. (D) Gab1 and Oct4 western blots of whole cell lysates of ESCs (ES), primary embryonic fibroblasts (fb), pre-iPS cells (pr), iPSCs reprogrammed in 2i medium (2i) or serum/LIF medium (sr) and an epiblast stem cell line (Epi). (E) Quantitative RT-PCR analysis of Gab1β and Gab1α expression in ESCs (ES) transitioned into epiblasts stem cells (Epi) and embryoid bodies (EB). Results represent means±s.d. from one experiment. Expression is normalised relative to the level in ESCs.

Stimulation of mouse ESCs via the gp130 cytokine receptor induces phosphorylation of the Gab1 adaptor protein (Burdon et al., 1999). However, the apparent molecular weight of Gab1 protein in mESCs, based on electrophoretic mobility, was significantly less than the 110-115 kDa reported for the Gab1 protein of differentiated somatic cells (Burdon et al., 1999; Holgado-Madruga et al., 1996; Weidner et al., 1996). To understand the basis of this difference, we compared Gab1 expression in mESCs with that in other embryonic cell types by western blotting and found that the predominant form of Gab1 protein in ESCs and embryonal carcinoma cells was ∼95 kDa, whereas embryonic fibroblasts and pooled tissues of a mid-gestation embryo possessed the typical 110-115 kDa form (Fig. 1A). Coordinated downregulation of a 4.5 kb Gab1 RNA and the 95 kDa Gab1 protein during embryoid body differentiation implied that the short-form Gab1 was encoded by this ESC-specific mRNA (Fig. S1A,B). We therefore sequenced Gab1 cDNAs obtained from an ESC cDNA library and found that all clones possessed a novel ∼50 nucleotide non-coding 5′ exon not present in the previously reported Gab1 cDNA, which is located ∼20 kb downstream from the first exon containing the established Gab1 translation start codon (Fig. 1B, Fig. S1C). Apart from the novel 5′ non-coding exon, the predicted protein coding sequences of the ESC Gab1 cDNAs was identical to that previously reported for mouse Gab1, suggesting that translation of the ESC Gab1 protein initiates at a methionine codon downstream of the normal Gab1 start codon. To identify this novel start codon, we transiently transfected COS7 cells with a series of Gab1 cDNAs where translation was initiated at each of the first four in-frame ATG codons. Initiation at the most 5′ methionine, M104, produced a Gab1 protein of similar mobility to that present in ESC protein lysates. (Fig. S1D). Methionine 104 is situated at the C-terminal boundary of the Gab1 PH domain (aa 10-117), and translation initiation from M104 eliminates most of this critical regulatory domain (Fig. 1C). The N-terminal PIP3 binding site that mediates membrane localisation, and a less well-characterised putative nuclear localisation sequence are also eliminated (Maroun et al., 1999b; Osawa et al., 2004). Nonetheless, the 95 kDa protein should retain all of the recognised direct protein-protein binding sites for partners such as Grb2, the MET receptor, PI3K and SHP2 and for our purposes will be referred to as Gab1β, to distinguish it from the previously characterised longer form, Gab1α.

To investigate how closely Gab1β expression was associated with the ESC state, we examined expression of the adaptor protein during induced pluripotent stem cell (iPSC) reprogramming of embryonic fibroblasts, and during exit from naive pluripotency to the ‘primed’ pluripotent state representative of post-implantation epiblast stem cells (EpiSCs). Western blot analysis showed that Gab1β was not expressed in partially reprogrammed pre-iPSCs (Fig. 1D), but was readily detected when cells transitioned to fully reprogrammed iPSCs derived either using standard serum/LIF, or the more stringent 2i condition that promotes the ‘naive’ ESC ground state (Boroviak et al., 2014, 2015; Nichols and Smith, 2009). In contrast, Gab1β expression was absent from EpiSCs derived from post implantation embryos (Fig. 1D). The association of Gab1β with the naive ESC state was confirmed by monitoring the levels of Gab1 transcripts during the transition of an ESC line through a stable EpiSC state and into differentiated embryoid bodies (Fig. 1E). Whereas expression of Gab1α increased as the ESCs transitioned to the primed state and then differentiated, Gab1β transcripts were sharply downregulated upon exit from the naive ESC state, in line with the loss of Gab1β protein expression from the post-implantation epiblast-derived cells (Fig. 1D). Interestingly, these switches in Gab1 transcription are also reflected in changes in epigenetic status of putative Gab1 promoters (Fig. S1E). In naive ESCs, H3K4me3 histone methylation associated with active promoters is enriched at the region immediately upstream of the Gab1β first exon, whilst the Gab1α promoter region, by contrast, is enriched for the repressive H3K27me3 mark commonly associated with gene silencing.

To determine whether Gab1β was expressed during preimplantation development *in vivo*, we performed RT-PCR amplification on mouse embryos and detected transcription of the novel 5′ exon of Gab1β in both fertilised oocytes and blastocysts (Fig. S2A*)*. Gab1β transcription was also detected in primordial germ cells and western blot analysis indicated a low level expression of the 95 kDa protein in the adult testis (Fig. S2A,B). By contrast, Gab1β protein was not readily detected in most other adult mouse tissues, suggesting that high level expression was primarily restricted to ESCs and germ cells (Fig. S2B).

Gab1 transcripts containing alternative 5′ exons have also been found in EST cDNA libraries in animals ranging from frogs to humans (Fig. S1C). The corresponding Gab1β exon in mouse was identified by cap-analysis of gene expression (CAGE), a technique that maps 5′ transcription initiation sites, providing evidence that a promoter within intron 1 drives expression of Gab1β (Fig. S2C). Transcription initiation at this site was also detected in trophoblast stem cell lines, suggesting that although this variant form of Gab1 protein is enriched in ESCs, expression may not be exclusive to pluripotent cells of the early mouse embryo.

### Gab1β promotes LIF-dependent signalling in ESCs

**Fig. 2.**
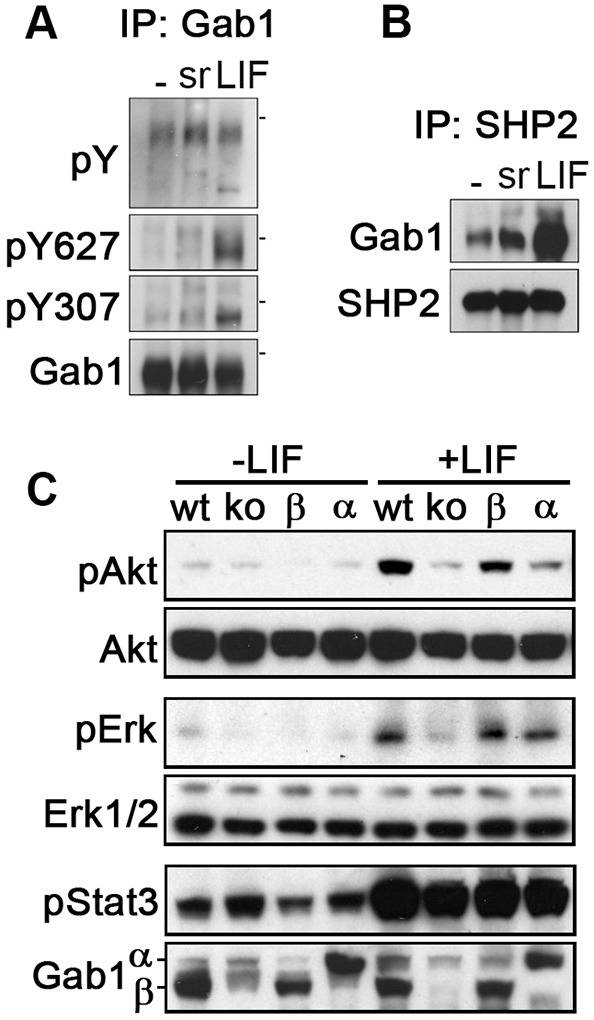
**Gab1β as an ESC signal transducer.** (A) Western blot of Gab1 immunoprecipitates from unstimulated ESCs (−), and ESCs stimulated with serum (sr) or LIF (lf) for 10 min, probed with Gab1pY627, Gab1pY307, phosphotyrosine and Gab1 specific antibodies. The dashes indicate position of 100 kDa MW marker. (B) Western blot of SHP2 immunoprecipitates from cells treated as in A probed with antibodies to Gab1 and SHP2. (C) Western blot of cell lysates from wild-type ESCs, and Gab1β knockout ESC line stably transfected with empty vector (−/−), Gab1β, and Gab1α expression vectors, unstimulated or following stimulation with LIF for 15 min. The blot was incubated with antibodies to phospho-Akt, Akt, phospho-Erk, Erk1/2, phospho-Stat3 and Gab1.

To evaluate the contribution of Gab1β to signalling in ESCs, we first examined tyrosine phosphorylation of Gab1β in response to the growth factor supplements LIF and foetal calf serum used in routine ESC cultures (Fig. 2A,B). Western blot analysis of Gab1 immunoprecipitates showed that the overall level of Gab1β tyrosine phosphorylation increased only slightly in response to LIF or serum. In contrast, however, tyrosine phosphorylation at specific SHP2 (Y627) and PLCγ (Y307) binding sites increased markedly in response to LIF, demonstrating that Gab1β is phosphorylated at recognised docking sites following stimulation of LIFR. Consistent with this phosphorylation pattern, SHP2 immunoprecipitates from LIF-induced ESCs contained Gab1β (Fig. 2B). Gab1β was also constitutively associated with the adaptor proteins p85 (the non-catalytic subunit of PI3K), Grb2 and ShcA in ESCs (Fig. S3A,B).

Without the PH domain to stabilise its association with the plasma membrane, Gab1β might not be able to effectively propagate downstream signals since, theoretically, the adaptor protein should be unable to participate in the PH-domain/PI3K/PIP3-dependent amplification mechanism (Rodrigues et al., 2000). Gab1β might even operate as a molecular sponge or decoy to dampen downstream effector functions. To determine how Gab1β influences downstream signalling we generated ESC lines that specifically lack Gab1β, but retain Gab1α expression. This was achieved by two consecutive rounds of homologous recombination with targeting vectors in which hygromycin- and blasticidin-resistance genes were inserted into the unique Gab1β 5′ exon (Fig. S3C,D,E).

To assess the role of Gab1β in LIF signalling, we examined phosphorylation of Akt, Erk and Stat3 in wild-type ESCs, in Gab1β KO ESCs, and in KO clones where we either restored Gab1β expression, or overexpressed Gab1α using stably integrated cDNA expression vectors (Fig. 2C). Whilst LIF-stimulated Stat3 phosphorylation was similar in all cell lines, the induction of Akt and Erk phosphorylation by LIF was noticeably reduced in Gab1β KO ESCs compared with levels in Gab1β wild-type ESCs. However, reconstitution of Gab1β or overexpression of Gab1α in Gab1β KO cells increased both Akt and Erk phosphorylation compared with levels in Gab1β KO cells. In contrast, Gab1β expression did not appear to play a significant role in serum-induced phosphorylation of Erk (Fig. S3F,G). These results demonstrated that despite not having a PH domain, Gab1β contributes to signalling downstream of LIFR in ESCs.

### Gab1β expression provides a growth advantage in high-density ESC cultures

To assess the biological function of Gab1β in ESCs, we first examined stem cell self-renewal, making use of the ESC-specific *Pou5f1-*βgeo knock-in allele in the IOUD2 parental ESC line, which allows the growth of undifferentiated stem cells to be monitored by measuring *Pou5f1*-*lacZ*-derived β-galactosidase activity (Mountford et al., 1994). We compared β-galactosidase activity in wild-type and Gab1β KO IOUD2 cells in self-renewal assays and found that the cell lines exhibited similar levels of self-renewal in response to different concentrations of LIF (Fig. S4A), indicating that Gab1β expression did not affect ESC self-renewal at the low cell densities used in these self-renewal assays.

**Fig. 3.**
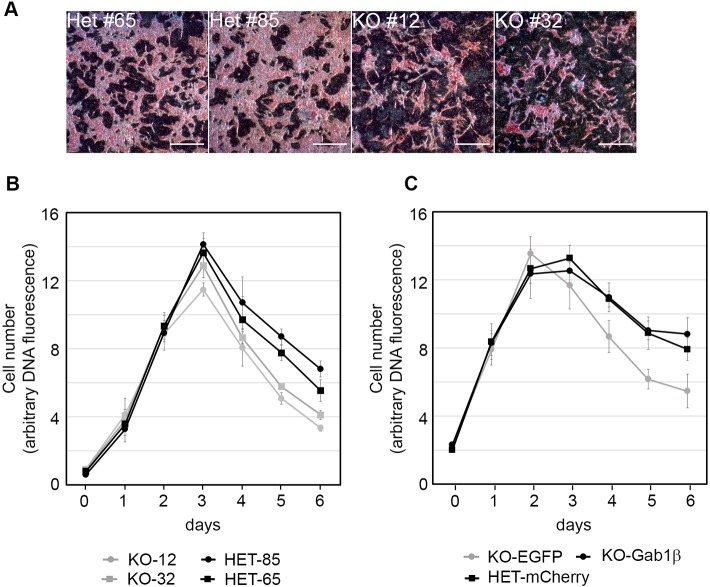
**Function of Gab1β in ESCs.** (A) Undifferentiated ESCs identified by alkaline phosphatase activity (pink stain) in two Gab1β heterozygous (HET) and two Gab1β knockout (KO) E14Tg2a cell lines 5 days after plating. Scale bars: 200 µm. (B) Growth curves of Gab1β heterozygous (HET) and Gab1β knockout (KO) E14Tg2a cell lines measured by DNA-dependent fluorescence in live cells. Cells were plated at a density close to that for routine passaging of ESCs (6×10^4^ cells/cm^2^) and DNA fluorescence was measured daily for 6 days. The data represent the mean±s.e.m. of three independent experiments. Statistical analysis using Student's *t*-test showed significant differences between the means for the HET and KO lines from day 3 to day 6 (*P*≤0.005). (C) Growth curves performed as described in B with pooled transfected ESCs: Gab1β heterozygous (HET-mCherry), Gab1β knockout (KO-EGFP) and Gab1β knockout stably expressing Gab1β from a cDNA expression vector (KO-Gab1β). Student's *t*-test analysis showed significant differences between the KO-EGFP and KO-Gab1β lines from day 4 to day 6 (*P*<0.0001), but not between HET-mCherry and KO-Gab1β cells.

To investigate whether Gab1β expression affects ESC growth at higher cell densities, we examined the behaviour of Gab1β-deficient ESCs in near confluent cultures. We used Gab1β mutant cell lines generated from the standard wild-type E14Tg2a parental cells (Fig. S4B,C), to exclude concerns that genetic modification of the *Pou5f1* gene might affect the response of cells in high-density assays (Karwacki-Neisius et al., 2013). Consistent with the previous IOUD2 experiments, self-renewal assays in the E14Tg2a Gab1β-expressing and KO lines did not reveal any consistent differences (Fig. S4D). When we plated two independent Gab1β heterozygous and two Gab1β KO E14Tg2a clones at densities typical of those routinely used for propagating ESC lines (∼10^5^ cells/cm^2^), the initial growth of the cell lines was indistinguishable in the first 2-3 days. However, once the lines approached confluence it appeared that the survival of Gab1β-expressing heterozygous cells was noticeably greater than that of the Gab1β KO cells (Fig. 3A). Monitoring ESC growth by measuring live cell DNA-dependent fluorescence daily throughout a 6-day culture period confirmed that the growth of all four clones was very similar for the first 2 days. However, between the 3rd and 4th days, when cell growth slowed, the Gab1β-expressing heterozygous cells attained higher cell numbers and maintained higher levels for an extended period (Fig. 3B). Statistical analysis showed that from day 3 onwards, the mean live cell DNA fluorescence differed significantly between the Gab1β heterozygous and KO cells. To confirm that this effect was due to Gab1β expression, we repeated the growth experiments using pools of Gab1β KO cells stably transfected with either a Gab1β expression vector or an EGFP control, and compared them with a pool of Gab1β heterozygous clones transfected with a mCherry expression control vector (Fig. 3C). As with previous experiments, the cell lines showed similar rates of growth during the first two days, but between day 3 and day 4, the Gab1β-restored and heterozygous cells exhibited a growth or survival advantage over the KO cells lacking Gab1β. There was a statistically significant difference between the live cell DNA fluorescence in EGFP control and Gab1β-expressing cells on days 4-6, but not between the Gab1β heterozygous mCherry-transfected cells and Gab1β-expressing cells (Fig. 3C). Collectively, these results establish that Gab1β expression provides a growth or survival advantage in high cell density culture conditions.

### Gab1β promotes ESC survival in nutrient-depleted conditions

**Fig. 4.**
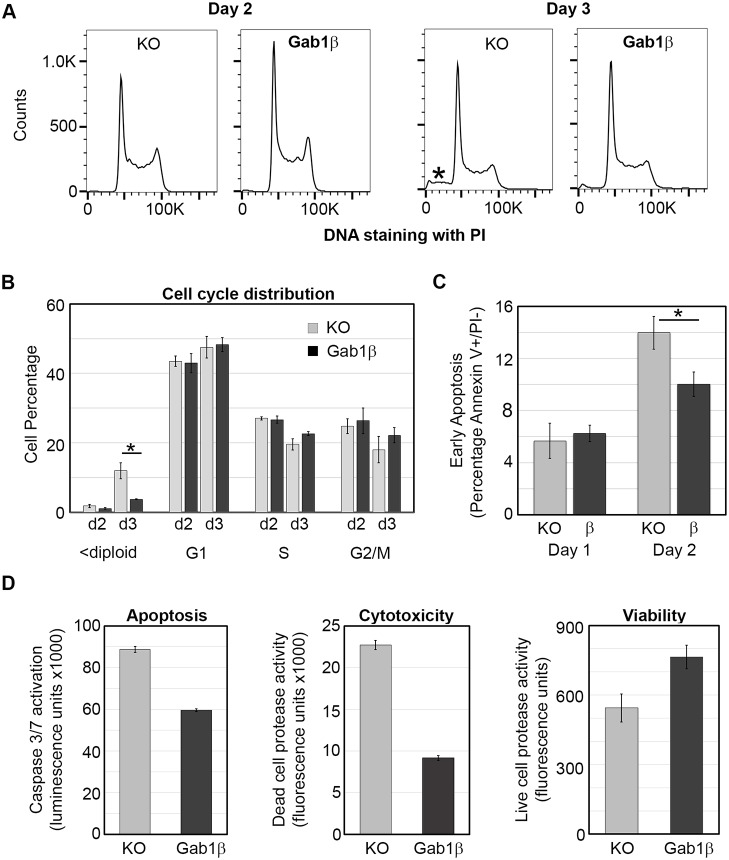
**Gab1β regulation of ESC cell cycle and apoptosis.** (A) Flow cytometry cell cycle analysis of Gab1β-expressing or non-expressing ESCs. Triplicate ESC samples were collected on day 2 and 3 of culture, fixed and stained using propidium iodide, and analysed by flow cytometry. Representative scans are shown and sub-diploid cell material is indicated with an asterisk. (B) Quantification of cell cycle distribution. Mean±s.d. values of cell cycle phases generated from three independent cultures (Student's *t*-test, *P<0.005). (C) Flow cytometry of Annexin V staining in day 1 and day 2 ESC cultures. Grey and black bars are values from Gab1β KO and Gab1β-expressing (Gab1β KO+Gab1β cDNA vector) ESCs, respectively (Student's *t*-test, *P<0.005). (D) Apoptosis, cytotoxicity and viability assays of day 2 cultures of Gab1β KO and Gab1β-expressing ESCs. Values are means±s.d. of four biological replicates (apoptosis and cytotoxicity *t*-tests, *P*<0.0001; viability *t*-test, *P*<0.005).

To determine the cause of the growth advantage associated with Gab1β expression, we examined cell proliferation and apoptosis in near-confluent ESC cultures. The cell cycle profiles of Gab1β-expressing and non-expressing cells were determined by measuring DNA content by flow cytometry. The overall profiles were indistinguishable during either the rapid or plateau growth phases, although as expected, there was a noticeable reduction in the S-phase contribution in both cell types during the plateau phase (Fig. 4A). However, it was evident that Gab1β KO cells accumulated a greater amount of sub-diploid/fragmented cells at day 3 during the plateau growth phase, pointing to increased levels of cell death in these cultures (Fig. 4A,B). We therefore used flow cytometry to assess the level of apoptosis by measuring the percentage of live cells that were positive for the apoptotic maker Annexin V. While equivalent levels of early apoptosis were observed at day 1 in both ESC cultures, at day 2 there was a marked increase in Annexin V staining in the Gab1β KO cells (Fig. 4C). Caspase 3/7 activation and cytotoxicity, reflecting general cell death, were both noticeably higher in the day 2/3 Gab1β KO cultures, further supporting the notion that absence of Gab1β was associated with increased levels of apoptosis (Fig. 4D). Taken together, these results suggest that expression of the Gab1β protein in ESCs promotes cell survival when culture conditions become restrictive.

**Fig. 5.**
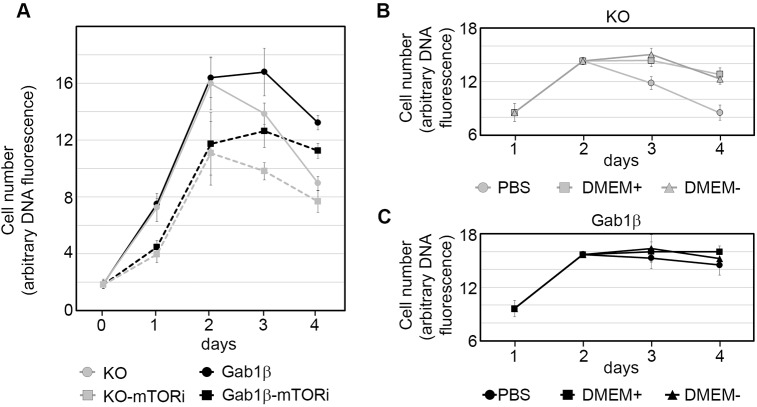
**Gab1β and ESC response to nutrient availability.** (A) Growth profiles of Gab1β KO and Gab1β-expressing ESCs treated with 25 nM mTOR inhibitor INK128 (mTORi). Values are means±s.e.m. of three independent experiments. (B,C) Growth of Gab1β KO and Gab1β-expressing ESC cultures supplemented on day 2 with 10 µl PBS, regular DMEM (+) or DMEM (−) lacking glucose, glutamine and sodium pyruvate. Values are means±s.d. of three biological replicates.

In high-density cultures a number of factors including environmental, physical or metabolic might limit ESC growth. To distinguish between these alternatives, we modified the culture environment and monitored the effects on ESC growth. To investigate what factors affect the growth profile, we treated ESCs with an inhibitor of the enzyme mTOR (mammalian/mechanistic target of rapamycin), a key enzyme that senses and controls nutrient availability in cells, and is a regulator of anabolic process such as protein, lipid and nucleotide synthesis (reviewed in Saxton and Sabatini, 2017). Addition of 25 nM mTOR inhibitor (INK128) restricted the initial growth rate of Gab1β-expressing and non-expressing cells equally and reduced the maximum cell numbers achieved in both cases (Fig. 5A). Under these restrictive conditions the growth or survival advantage of the Gab1β-expressing cells was still apparent, even though it occurred at lower cell numbers, implying that it was unlikely that cell density was responsible for limiting the later phase of ESC growth.

To determine whether nutrients might be limiting the growth of Gab1β-deficient ESCs, we tested how supplementation with medium components affected ESC culture growth and survival at the plateau phase (Fig. 5B,C). Addition of either standard DMEM, or DMEM lacking glucose, sodium pyruvate and glutamate at day 2 of culture improved the survival of Gab1β KO ESCs, suggesting that depletion of amino acids or vitamin supplements common to both growth media become limiting for ESC growth during the plateau phase, and excluded the depletion of energy sources such as glucose as being directly responsible for slowing ESC growth. Direct supplementation of plateau phase cultures with glutamine or sodium pyruvate also did not appreciably improve ESC survival or growth (Fig. S5).

### Gab1β-mediated ESC survival is LIF dependent

**Fig. 6.**
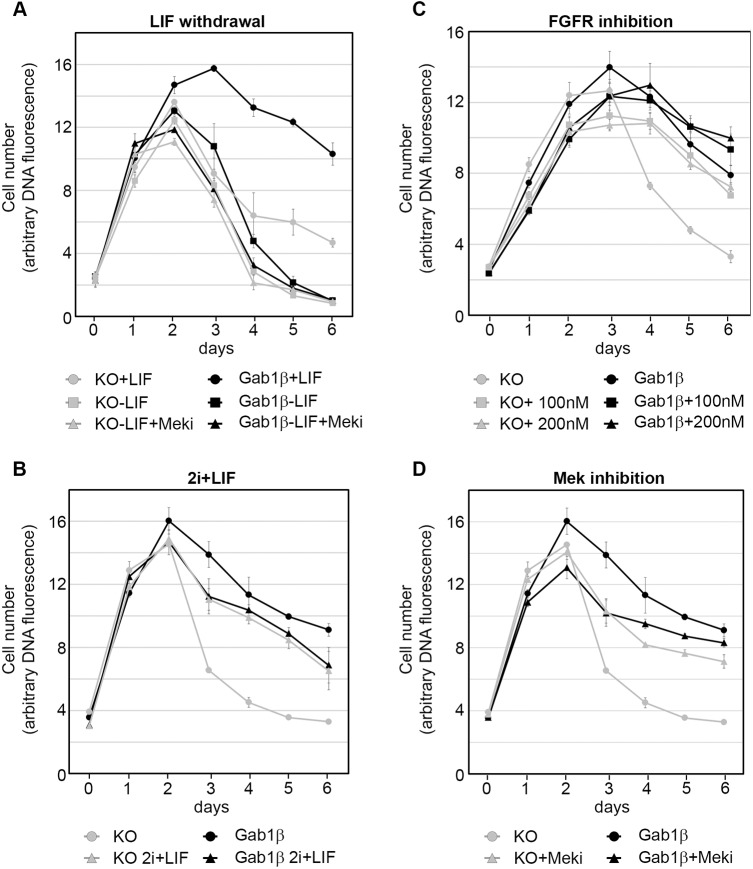
**Gab1β-mediated ESC survival depends on LIF, and is rescued by Mek inhibition.** Growth profiles of Gab1β KO and Gab1β-expressing ESCs cultured in the presence or absence of: (A) LIF (±1 µM PD0325901 Mek inhibitor); (B) 2i (1 µM PD0325901 Mek inhibitor, 3 µM CHIR99021 GSK3 inhibitor) +LIF serum free medium; (C) FGFR inhibitor PD173074 (one experiment with quadruplicate biological samples; and (D) 1 µM PD0325901 Mek inhibitor. Data points in A,B and D represent the means±s.e.m. of three independent experiments Data points in graph C represent the means from four biological replicates in one experiment. For all graphs, Student's *t*-tests showed statistically significant differences between serum+LIF-treated KO and Gab1β-expressing control lines at days 4-6 (*P*≤0.0001), and between KO control and KO treated (KO+Meki) cells (*P*≤0.0001).

We have shown that Gab1β is phosphorylated after stimulation of the LIFR, and activates downstream signalling (Fig. 2). To determine whether LIF signalling was also required for Gab1β-mediated ESC survival in nutrient-depleted cultures, we compared the growth of Gab1β-expressing and non-expressing cells with or without LIF over 6 days. Whereas cells in all conditions grew rapidly for the first 2 days, in both Gab1-expressing and KO cultures deprived of LIF, cell numbers declined rapidly after this point in a manner similar to that displayed by Gab1β KO cells cultured in the presence of LIF (Fig. 6A). This demonstrated that ESC survival in post-confluent cultures relies on LIF signalling and also depends on Gab1β expression. Although unlikely, it is possible that the loss of cell viability seen here is a result of ESC differentiation initiated by LIF withdrawal. To address this possibility, we restricted ESC differentiation by including the Mek (MEK1/2; also known as MAP2K1 and MAP2K2) inhibitor PD0325901 in the LIF-deficient high-density cultures. However, this did not improve ESC survival (Fig. 6A), and may even have compounded the effects of LIF withdrawal. To explore the relationship between Gab1β and LIFR-mediated cell survival, we examined the LIF dose response of ESC cultures (Fig. S6A,B). Whereas the survival of the Gab1β-expressing cells at the plateau phase was dependent on the dose of LIF, the acute collapse in viability of the majority of the Gab1β KO cells was largely independent of the dose.

### Survival of Gab1β KO ESCs is rescued by inhibition of FGFR or Mek activity

The studies above were performed under standard serum+LIF culture conditions, but we were interested in determining how Gab1β expression might affect ESC growth and survival under culture conditions, such as 2i+LIF, that selectively promote the naive ESC state. We found that while cell proliferation in the initial growth phase was similar to that in serum-containing medium (Fig. 6B), in the post-plateau phase, Gab1β KO cell survival in 2i+LIF medium was markedly better than in serum+LIF medium, and comparable to cells expressing Gab1β. There were differences in growth characteristics of ESCs in 2i+LIF serum-free and serum+LIF medium, with 2i+LIF cells forming more compact and tighter colonies. Nonetheless, these results indicate that conditions induced by 2i+LIF compensate for the absence of Gab1β in the Gab1β KO cells. To determine how 2i+LIF contributes to this ‘rescue’ effect, we inhibited either Mek or its upstream activator FGFR (fibroblast growth factor receptor) in the standard serum+LIF conditions. Treatment of cells with Mek inhibitor or the FGFR inhibitor PD173074 had little effect on the initial rate of expansion of ESCs but in both cases improved the survival of the Gab1β KO cells (Fig. 6C,D). Taken together, these results indicate that the survival function of Gab1β and inhibition of FGFR- and Mek-dependent activities converge to promote ESC survival.

### Gab1β is located at ESC membranes

**Fig. 7.**
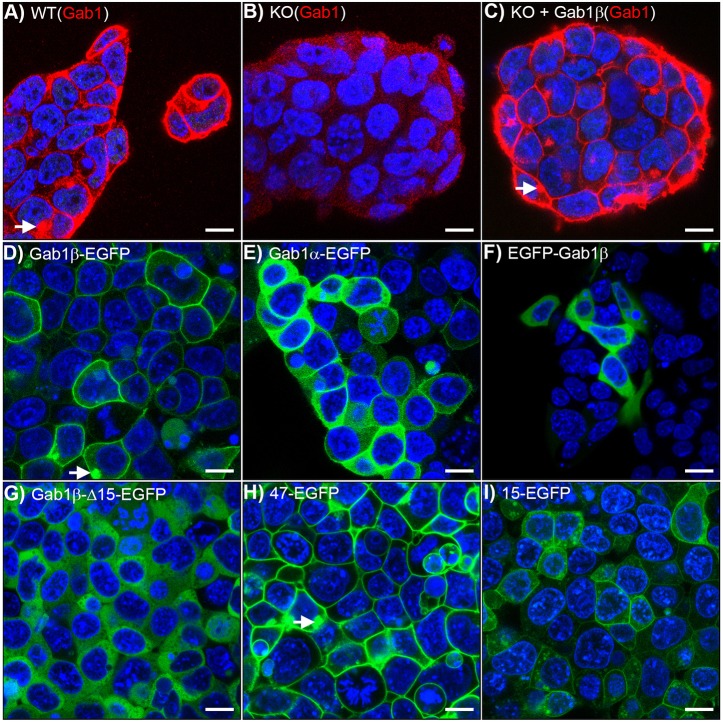
**Localisation of Gab1β at the cell membrane.** Confocal images of (A) wild-type ESCs, (B) Gab1β knockout ESCs and (C) Gab1β knockout ESCs stably transfected with a Gab1β cDNA expression vector, immunostained with antibodies against Gab1 (red) and counterstained for DNA with DAPI (blue). (D-I) Confocal images of EGFP fluorescence in ESC stably expressing the following Gab1-EGFP fusion proteins: (D) Gab1β-EGFP; (E) Gab1α-EGFP; (F) N-terminal EGFP-Gab1β; (G) Gab1β-EGFP fusion lacking 15 N-terminal amino acids; (H) the 47 N-terminal amino acid peptide of Gab1β fused to EGFP; and (I) 15 N-terminal amino-acid peptide of Gab1β fused to EGFP. White arrows highlight consistently observed intracellular accumulations of Gab1β proteins. Scale bars: 10 µm.

Localisation of Gab1 at the cell membrane is thought to be critical for its functional activity and yet, despite lacking an intact PH domain, Gab1β contributes to ESC signalling and promotes ESC survival. To understand how Gab1β could influence ESC survival without the PH domain, we examined the cellular location of Gab1β in ESCs. Based on previous reports, a Gab1 protein lacking the phospholipid binding site contained within the PH domain should be located in the cytoplasm (Maroun et al., 1999b; Rodrigues et al., 2000). However, when we compared the location of Gab1 protein in wild-type and Gab1β KO ESCs using immunocytochemistry (with the antibody that recognises both Gab1α and -β forms), it was clear that a significant proportion of Gab1 protein localised to the cell membrane in wild-type cells (Fig. 7A). By contrast, in Gab1β KO cells there was very little membrane-associated Gab1 protein (Fig. 7B). Crucially, stable expression of a Gab1β cDNA in the KO cells restored the wild-type distribution of Gab1, demonstrating that Gab1β in ESCs is normally located at the cell membrane (Fig. 7C).

To determine how Gab1β is directed to the membrane, we examined the cellular localisation of Gab1-EGFP fusion proteins stably expressed in the Gab1β KO cells. Whereas the Gab1β-EGFP fusion protein carrying EGFP at the C-terminus of Gab1β was located predominantly at the ESC membrane (Fig. 7D), the corresponding Gab1α-EGFP protein was largely cytoplasmic, with only a minority of ESCs displaying membrane-associated Gab1α-EGFP (Fig. 7E). Significantly, a fusion protein in which EGFP was attached to the N-terminus of Gab1β was entirely cytoplasmic (Fig. 7F). This result suggested that the membrane-targeting signal of Gab1β can be blocked by attachment of EGFP, or the PH domain, and is therefore likely to be located close to the N-terminus of the protein.

Gab1 proteins that lack the entire PH domain (amino acids 10-117) have been shown to be located in the cytoplasm (Maroun et al., 1999b; Rodrigues et al., 2000). However, these artificial mutants are not directly comparable to Gab1β-expressing cells as we noted that the N-terminus of Gab1β retains an additional 15 amino acids (15 aa) from the end of the PH domain. This 15 aa polypeptide forms a structurally conserved amphipathic helix that typically caps off PH domains (Lemmon and Ferguson, 2000). To examine the contribution of this 15 aa polypeptide, we deleted this element from the Gab1β-EGFP fusion protein and found in agreement with previous reports (Maroun et al., 1999b; Rodrigues et al., 2000) that the Gab1β-Δ15aa-EGFP protein was located in the cytoplasm (Fig. 7G). To examine directly how the N-terminal region of Gab1β contributes to membrane targeting, we examined the localisation of EGFP fusion proteins carrying the N-terminal 47 amino acids (aa 104-150) or just the 15 amino acid peptide (aa 104-118) of Gab1β (Fig. 7H,I). The 47aa-EGFP fusion protein was very clearly located at the membrane. In comparison, the 15aa-EGFP fusion protein exhibited a weaker signal at the membrane and evidence of an irregular distribution throughout the cytoplasm. Taken together, these experiments suggest that the N-terminal region of Gab1β, containing the residual 15 aa α-helical region derived from the PH domain, is sufficient to target Gab1β to the ESC membrane. Gab1β protein also accumulated at an intracellular site in most ESCs (Fig. 7A,C), as did the Gab1β-EGFP and 47aa-EGFP fusion proteins (Fig. 7D,H), indicating that Gab1β may also associate with the membranes of intracellular vesicles.

### Palmitoylation of Gab1β drives its membrane localisation and function

**Fig. 8.**
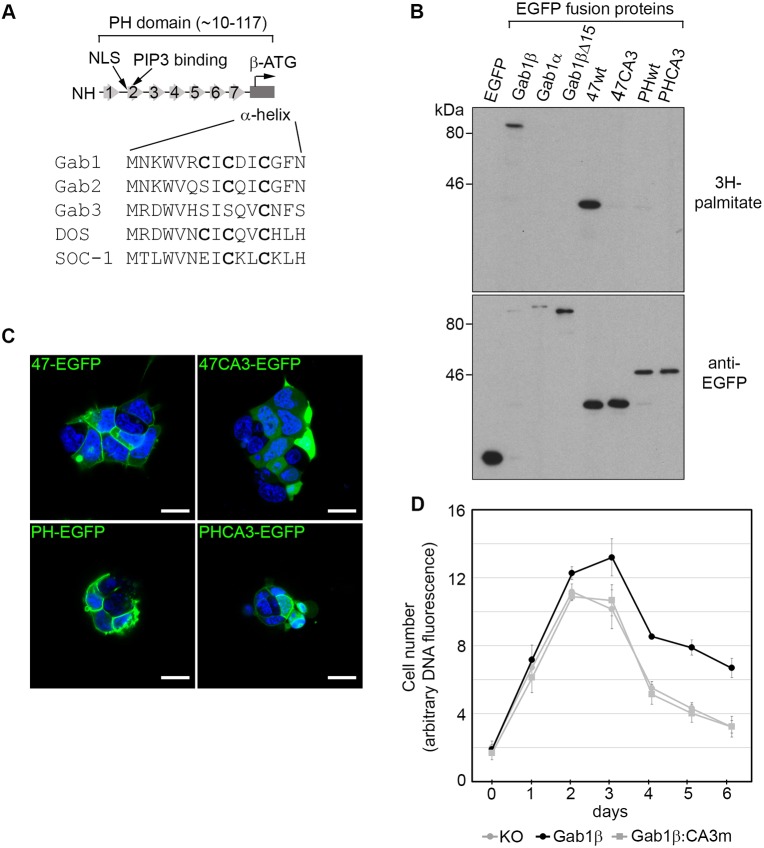
**The N-terminus of Gab1β directs palmitoylation and is required for membrane localisation and function.** (A) Schematic outlining the structure of the Gab1 PH domain and comparison of the N-terminus of Gab1β with corresponding regions of other Gab-related proteins. The arrangement of the seven β-sheets and the α-helix, and relative positions of a putative nuclear localisation sequence (NLS) and PIP3 binding residues are shown. Cysteines within the α-helix are highlighted in bold. (B) EGFP immunoprecipitates from ESCs stably transfected with EGFP or Gab1-EGFP fusion proteins cultured overnight with [^3^H]palmitate, were fractionated on an SDS protein gel, transferred to a filter and autoradiographed (top) and probed for EGFP protein (bottom). CA3 designates variants in which the three N-terminal cysteines of Gab1β are substituted with alanine. (C) Confocal images of EGFP fluorescence in ESCs transfected with the 47-EGFP, 47CA3-EGFP, PH-domain EGPF and PH-domain CA3-EGPF fusion proteins (counterstained with DAPI). Scale bars: 10 µm. (D) Growth curves of Gab1β knockout (KO) ESCs and Gab1β KO ESCs stably transfected with either Gab1β wild-type or CA3 mutant cDNA expression vectors. The graphs represent the means±s.e.m. obtained from three independent experiments. Student's *t*-test analysis showed significant differences between the KO and Gab1β-expressing lines from day 4 to day 6 (*P*<0.0001), but not between KO and KO-Gab1β:CA3m cells.

Our localisation experiments demonstrate that the 15 aa N-terminus of Gab1β contains a signal that targets it to ESC membranes. Examination of this region identified three cysteines that were potential sites for palmityolyation: a lipid modification that could account for the stable association of Gab1β with the cell membrane (Fig. 8A). The three cysteines are conserved to different extents amongst Gab family members, including the Gab1 homologues DOS in *Drosophila* and SOC-1 in *Caenorhabditis*. To test directly whether Gab1β is palmitoylated in ESCs, we cultured ESCs expressing Gab1-EGFP fusion proteins in the presence of [^3^H]palmitate and examined ^3^H-labelling of EGFP immunoprecipitates after electrophoresis and transfer to an immobilising filter. Autoradiography revealed that Gab1β and the 47 aa fusion constructs were labelled efficiently with [^3^H]palmitate (Fig. 8B). By contrast, deletion of the 15aa region from Gab1β, or alanine substitution of the three cysteines in the 47 aa fusion protein (47aa-CA3m-EGFP), prevented labelling. Interestingly, ^3^H-labelling of Gab1α or Gab1 PH-domain–EGFP fusion proteins could not be detected, demonstrating that in the presence of the intact PH domain Gab1 palmitoylation is suppressed or at least was inefficient under these experimental conditions. To determine how palmitoylation of Gab1 affects membrane targeting, we examined the localisation of the 47aa-CA3m-EGFP in ESCs and found that alanine substitution of the three cysteines in this mutant abolished membrane targeting of the fusion protein (Fig. 8C). Significantly, the identical alanine substitutions within the context of the PH-domain–EGFP fusion protein did not prevent localisation at the membrane (Fig. 8C).

These results strongly suggest that palmitoylation of the N-terminal domain drives membrane localisation of Gab1β in ESCs. We therefore determined whether palmitoylation of Gab1β was also associated with supporting ESC survival. We compared the growth of Gab1β KO ESCs with cells stably expressing cDNAs encoding either Gab1β or a Gab1β-CA3 mutant. In contrast to cells expressing wild-type Gab1β, ESCs expressing the Gab1β-CA3 mutant protein exhibited the same growth profile as the Gab1β KO cells, showing that the palmitoylation of Gab1β and localisation at the ESC membrane is required for Gab1β-dependent ESC survival in culture (Fig. 8D).

## DISCUSSION

LIFR-gp130 signalling has essential roles during early embryonic development that provide the physiological rationale for the contribution of this signalling pathway to the growth and self-renewal of embryonic stem cells (ESCs) in culture (Do et al., 2013; Nichols et al., 2001). Here, we report the identification of a novel variant form of the adaptor protein Gab1 (Gab1β) that contributes to LIF-dependent survival of ESCs under conditions of limited nutrient availability. In this Gab1 variant the domain that normally regulates the access of the protein to the membrane and to activated receptors is replaced with a lipid tag that anchors the Gab1β adaptor protein constitutively at the cell membrane. Although disruption of *Gab1* expression does not normally disturb pre-gastrulation embryo development, we presume that the switch in regulatory modes may confer an additional growth or survival advantage under exceptional circumstances (Itoh et al., 2000; Sachs et al., 2000). Since Gab1β is expressed in cells representative of the early preimplantation epiblast and the trophoblast stem cell compartment, we speculate that a primary function of Gab1β is to support preimplantation embryos exposed to suboptimal environmental conditions in the uterus.

The abundance of Gab1β protein and its phosphorylation following treatment with LIF suggested that the adaptor protein has a functional role in ESCs. Under the standard clonal culture conditions typically used to assess growth and self-renewal, ESCs tolerated the loss of Gab1β. However, when challenged with nutrient-limited conditions, Gab1β expression reduced cell death and extended the viability of ESCs. When cells were treated with an inhibitor of the key nutrient sensor and anabolic regulatory enzyme mTOR, Gab1β expression improved ESC survival, which could imply that availability of nutrients is a contributory limiting factor. Indeed, rescue of starved ESC cultures by supplementation with minimal basal medium indicated that lack of either amino acids or vitamins was responsible for collapse of the cultures. It has been reported that restricting ESC growth induces a state that resembles the quiescent condition induced in embryos during delayed implantation (also known as diapause) (Renfree and Shaw, 2000). *In vivo*, preimplantation blastocysts undergo developmental arrest and implantation is delayed in order to synchronise embryo development with the mother's reproductive capacity and environmental inputs. Importantly, inhibition of mTOR is reported to suppress ESC growth and to maintain a ‘paused’ quiescent, but developmentally competent, state in ESCs and blastocysts for over a week (Bulut-Karslioglu et al., 2016). This suggests that nutrient availability may determine entry into this quiescent condition and this state can be induced experimentally in culture.

A role for Gab1β in ESC survival was evident in LIF-treated cultures. Gab1β expression also increased LIFR-mediated Erk and Akt phosphorylation, directly implicating the adaptor as a transducer of LIFR signals. PI3K/Akt signalling has been shown to be involved in Gab1-mediated cell survival, and in ESCs, activation of this pathway has been reported to promote cell growth, survival and self-renewal (Cherif et al., 2015; Fan et al., 2016; Fukumoto et al., 2009; Furuta et al., 2016; Hishida et al., 2015; Holgado-Madruga and Wong, 2003; Holgado-Madruga et al., 1997; Jirmanova et al., 2002; Paling et al., 2004; Sun et al., 2014; Watanabe et al., 2006). By contrast, Erk activation is normally associated with ESC differentiation, and inhibition of Mek-Erk signalling promotes ESC self-renewal (Burdon et al., 1999; Ying et al., 2008). Notably, inhibition of either FGFR or Mek activity improved the survival of Gab1β-KO ESCs, and appeared to largely rescue the Gab1β deficiency. Indeed, it has been reported that Mek inhibition reduces the requirement for PI3K signalling in ESCs, thus potentially uncoupling cells from the requirement for PI3K-dependent survival signals provided by Gab1β (Hishida et al., 2015). A possible alternative explanation is that Gab1β might be a physiological disruptor of Mek signalling by altering the activation kinetics of the pathway, or by binding Erk and regulating access of this kinase to its target proteins (Osawa et al., 2004; Wolf et al., 2015).

Membrane localisation of Gab1β is essential for its survival function and is dependent on palmitoylation of this ESC adaptor protein. The lipid modification targets Gab1β to ESC membranes, liberating Gab1β from dependence on PI3K activity and availability of the phospholipid PIP3, and locates the adaptor in close proximity to upstream effectors such as receptors and associated kinases. Removal of the PH domain also eliminates an auto-inhibitory interaction between the PH domain and C-terminal region of Gab1 (Eulenfeld and Schaper, 2009; Wolf et al., 2015). This blocks PIP3 binding by the PH domain, but can be relieved by Erk-mediated phosphorylation of the C-terminal domain. Freed from this regulatory constraint, Gab1β should be constitutively available to associate with receptors and maintain the basal level of Gab1β phosphorylation observed in ESCs. Nonetheless, Gab1β cannot participate in the PH-domain/PI3K/PIP3 positive feedback loop and therefore will be limited in its capacity to amplify downstream signalling. Understanding why Gab1β expression is preferred in ESCs rather than upregulation of Gab1α expression should provide insights into how quantitative and qualitative aspects of signalling affect cell behaviour in the early embryo. Perhaps direct association of Gab1β with the cell membrane increases sensitivity to LIFR-gp130-mediated activation, but limits the intensity and duration of signalling to curb differentiation or proliferation – thus supporting stem cell survival under growth-restrictive conditions.

Gab variants with alternative N-terminal sequences have been described previously. A hamster Gab1 protein (Gab1^Δ1-103^) analogous to Gab1β, enhances anchorage-independent growth of preneoplastic fibroblasts (Kameda et al., 2001), and PH-domain-deficient isoforms have also been reported for the closely related Gab-family member Gab2 (Adams et al., 2012; Gu et al., 1998, 2001). Interestingly, membrane localisation of another IRS/DOS family member, fibroblast growth factor receptor substrate 2 (FRS2) depends upon myristoylation to target this adaptor protein to the cell membrane and enable its participation in downstream signalling (Kouhara et al., 1997). Gab protein variants may therefore exemplify a more general mechanism for diversifying the intracellular locations of this class of adaptor proteins and their contribution to signalling.

Gab1β is highly expressed in ‘ground state’ naive ESCs that are thought to closely represent the epiblast of the preimplantation embryo (E3.75-E4.5) (Boroviak et al., 2014). Interestingly, although Gab1β expression is down-regulated during the transition to form EpiSCs, a cell type representing the post-implantation epiblast (Brons et al., 2007; Tesar et al., 2007), high levels of Gab1β transcription are also detected in trophoblast stem cells. This may indicate that Gab1β transcription is regulated by factors common to both epiblast and trophoblast stem cells, and additionally point to Gab1β having a broader role in regulating the viability of cells within the whole preimplantation embryo.

In conclusion, Gab1β is highly expressed in rodent ESCs and supports LIF-dependent survival when nutrient availability becomes limiting. How Gab1β and its downstream effectors interact with the regulatory machinery of cells in the preimplantation embryo, and their relevance to conditions prevalent *in vivo* deserves further consideration and could provide new insights into the control of embryo survival.

## MATERIALS AND METHODS

### Cell culture and transfections

ESCs were cultured without feeder cells in Glasgow modification of Eagle's medium (GMEM) containing 10% foetal calf serum, 0.1 mM 2-mercaptoethanol and LIF as described previously (Chambers et al., 2003; Hooper et al., 1987; Mountford et al., 1994; Niwa et al., 2000; Smith, 1991). R2 pre-iPS cells were cultured on irradiated DIAM feeders (Meek et al., 2010) in GMEM/FCS (Theunissen et al., 2011). iPS cells were cultured in 2i+LIF medium (Theunissen et al., 2011) or with GMEM/FCS/LIF as described for ESCs above. EpiSC cells were grown on fibronectin-coated plates in N2B27 medium containing activin (20 ng/ml) and FGF2 (12 ng/ml) (Guo et al., 2009; Osorno et al., 2012). PSMB embryonal carcinoma, primary mouse embryonic fibroblasts, COS7, NIH 3T3 and C3H 10T1/2 cells were maintained in ESC culture medium without LIF. For routine ESC culture, LIF was generated in-house. For inductions and growth experiments, ESGRO recombinant mouse LIF protein (ESG1107) was obtained from Merck. The small-molecule inhibitors PD0325901 (#1408) and CHIR99021 (#1386) were obtained from Axon Medchem, and PD173074 (S1264) from Selleckchem.

COS7 were transfected using the Fugene 6 transfection reagent in accordance with the manufacturer's instructions (Roche Molecular Biochemicals) at ∼50% confluence, were incubated for 72 h with 2 µg of supercoiled plasmid DNA complexed with 5 µl Fugene and then harvested for protein analysis. Transfected ESCs were obtained by incubating the cells (0.5-1×10^6^ cells per well of 6-well dish) with 5 µg of plasmid DNA using Lipofectamine 2000 reagent (Life Technologies) for 36 h. ESC clones stably transfected with Gab1 expression vectors were selected in ESC medium containing puromycin (1 µg/ml) or hygromycin (100 µg/ml) (for 10 days, then trypsinised and combined to establish pooled cultures for each construct. For growth factor induction experiments, ESCs were cultured overnight in GMEM base medium lacking glutamine and serum prior to stimulation with growth factors. E14/T cells were super-transfected with supercoiled plasmids carrying the polyoma origin of replication, as described previously.

### Cloning of Gab1 cDNAs and expression constructs

The mouse Gab1α cDNA was generated by RT-PCR using a Superscript preamplification system (Invitrogen) to reverse transcribe 1 μg of total RNA from C3CH10T1/2 fibroblasts. 1/10th of the reaction was amplified using Expand High fidelity polymerase (Roche) with 5′ GGGCGGCCGCCGCACC**ATGAGCGGTGGTGAAGTG** and 3′ CCCTCGAG**TCACTTCACATTCTTGGTGGGTG** oligonucleotide primers containing *Not*I and *Xho*I restriction sites, respectively (underlined). Thirty cycles of PCR generated the expected 2 kb DNA fragment, which was restricted with *Not*I and *Xho*I, subcloned and sequenced. Gab1β cDNAs were isolated from an ESC cell cDNA library provided by Dr Hitoshi Niwa ( Institute of Molecular Embryology and Genetics, Kumamoto, Japan). Three independent clones were sequenced at their 5′ and 3′ ends and one clone sequenced on both strands of the coding region. Gab1α and Gab1β cDNAs were restricted with *Not*I and *Xho*I and subcloned into pCAGIH a hygromycin-resistant derivative of the pCAGIZ expression vector (Jackson et al., 2002; Niwa et al., 1998). This plasmid contains the SV40 origin sequence, allowing efficient transgene expression in cells harbouring the SV40 large T antigen. A progressively truncated series of Gab1 cDNAs, M104, M151, M232 and M240, were generated by PCR amplification. Reactions were performed with a 5′ primer containing a *Not*I restriction site and Kozak consensus immediately upstream of the ATG initiation codon plus 16-19 nucleotides of downstream Gab1 sequence and a 3′ primer containing a *Xho*I restriction site. The amplified products were subcloned into the pCAGIH expression vector as *Not*I/*Xho*I fragments. Gab1-EGFP- or Gab1-myc-tagged fusion proteins were generated by PCR amplification of Gab1 regions cloned in pCAGIH (or puro version pCAGIP) modified vectors, upstream of an open reading frame containing either a poly glycine-EGFP protein, or a triple myc-tag, respectively. The 15 aa N-terminal region of Gab1β was cloned upstream of EGFP as a double-stranded oligonucleotide. The sequences of the oligonucleotide primers used to generate the Gab1 coding regions are available on request. Gab1 expression vectors were linearised with *Sfi*I prior to transfection. Drug-resistant ESC colonies were picked individually or pooled to establish stably transfected cultures.

### Preparation of embryonic RNA and RT-PCR analysis

Embryos and cells were prepared from appropriately staged strain 129 female mice. After flushing from the oviduct, oocytes were treated with hyaluronidase to remove cumulus cells. Both oocytes and blastocysts were washed extensively to eliminate contaminating cellular debris prior to lysis. Primordial germ cells (PGCs) were isolated from the dissected genital ridges of day 12.5 embryos, using calcium- and magnesium-free phosphate buffered saline (Buehr and McLaren 1993). Freshly prepared oocytes (*n*=35), blastocysts (*n*=30) and PGCs were lysed in Solution D and RNA was purified by acid phenol extraction (Chomczynski and Sacchi, 1987). To aid recovery of RNA, 20 µg of carrier tRNA was added to the samples prior to extraction. cDNA was prepared from 1/5th of the recovered RNA using random priming and the Superscript Preamplification system. 1/10th of the reverse transcription reaction was then amplified using AmpliTaq Gold polymerase (Perkin Elmer). PCR reactions of 50 cycles were performed using primers that amplify a 184 bp DNA fragment from Gab1β cDNA (GGACCATTCGAGGTGGCAGAC; CAACCCAGCATCAACTTGCTGAC) or a 939 bp DNA fragment from β-actin cDNA (GTGACGAGGCCCAGAGCAAGAG; AGGGGCCGGACTCATCGTACTC).

### Disruption of Gab1β expression by homologous recombination

Genomic DNA spanning the Gab1β exon was obtained by screening the RPC121 Mouse PAC library obtained from UK HGMP Resource Centre (Osoegawa et al., 2000) with the ^32^P end-labelled Gab1β oligonucleotide TACTCAGGTGTCATGCGTCTGCCACCTCGAATGGT. A ∼7 kb *Xba*I DNA fragment encompassing the Gab1β exon was isolated from the PAC clone 340-d21, cloned into pBS and sequenced using the Genome Priming System (New England Biolabs). ET-cloning using bacterial recombination was used to introduce a kanamycin gene into the Gab1β exon to create a unique *Bam*HI site into which a PGK polyadenylation signal was cloned (Muyrers et al., 1999). This sequence was amplified with a 5′ primer incorporating *Bam*HI and *Sal*I restriction sites and 3′ primer containing a *Bgl*II site. Hygromycin, and blasticidin selection markers were then individually cloned as *Bam*HI, *Sal*I fragments immediately upstream of the PGK sequence to generate two targeting vectors. Plasmid was digested with *Xba*I, to free the targeting vector from the plasmid backbone, prior to transfections. In transfections, either 1×10^8^ ESCs were electroporated (800V, 3 µF) with 150 µg linearised plasmid or 1×10^7^ cells were electroporated (240V, 500 µF) with 40 µg linearised plasmid. Cells were plated at 2-3×10^6^ cells per 10 cm dish and 48 h later treated with medium containing blasticidin (10 µg/ml), hygromicin (100 µg/ml) or G418 (200 µg/ml). After ∼10 days of selection, colonies were picked and expanded for freezing and DNA analysis. Genomic DNA was prepared from clones grown to confluency in 24-well plates using proteinase K digestion followed by isopropanol precipitation. Approximately 1/5th of the resuspended DNA was restricted with *Eco*RV, transferred to an uncharged Nylon filter (Amersham) by Southern blotting and hybridised with DNA probes, located either 5′ or 3′ of the targeting construct, generated by PCR (5′ probe primers: 5′-AGAGTCCTGTTGTATGCCTGG-3′, 5′-CAAGTACTCCTTACTGCCCAG-3′; 3′ probe primers: 5′-GACTCACCAGAAATGGGGTTC-3′, 5′-AGGTGATGTGGTTTCATGTAG-3′).

### ESC self-renewal assays

To compare stem cell self-renewal between wild-type and Gab1 mutant IOUD2 cells, Oct4-β-galactosidase activity was quantified as described previously (Burdon et al., 1999). ESCs were plated in 24-well plates (5000 cells/well), cultured for 6 days and β-galactosidase activity in lysates was measured using the ortho-nitrophenyl-β-galactoside (ONPG) assay. Specific enzyme activity was normalised relative to the protein concentration of lysates and standardised against a serial dilution of purified β-galactosidase enzyme (Promega). Triplicate samples were assayed as duplicates.

Alkaline phosphatase activity was assayed in ESC lysates prepared from cells in 24-well plates (described above), using 1 ml of 1 mM MgCl_2_, 0.2% NP-40 per well and incubated for 2 h at 37°C. Duplicate aliquots (80 µl) were mixed with 10 µl glycine buffer (1 M glycine, 10 mM ZnCl_2_, 10 mM MgCl_2_), and 10 ml of 100 mM p-nitrophenyl phosphate (Sigma), incubated in a 96-well plate for 20-60 min and the absorbance was read at 405 nm.

### Cell growth and proliferation assay

Cells were plated onto uncoated tissue culture 96-well plates at 20,000 or 40,000 cells per well in 100 µl of growth medium, and cell growth was assayed using the CyQuant Direct Cell Proliferation Assay (C35011 ThermoFisher Scientific) to measure live cell-associated DNA fluorescence. Each sample, was assayed by addition of 100 µl of 2× detection reagent (0.4 µl Direct DNA stain, 2 µl Direct Background suppressor diluted in 100 µl Opti-MEM ThermoFisher Scientific 11058021), incubated for 1 h at 37°C in the incubator and then read on Victor Multi Label Counter from the bottom of plates with standard green filter at 508/527 nm excitation/emission wavelengths. Each cell line sample or treatment was measured in a minimum of four independent wells for each experiment.

### Cell cycle analysis

Single cell suspensions of cells were fixed in 70% ethanol, rehydrated in phosphate buffered saline for 30 min at 4°C, rinsed twice with PBS and then incubated with 100 µg DNAse-free RNAse for 1 h at room temperature. Propidium iodide (final concentration 50 µg/ml) was added to the cells for 10 min before flow analysis (Fortessa X-20, Becton Dickinson: 561 nm laser, emission 610 nm). Data were acquired using FACSDiva software (Becton Dickinson). The cytometer was set to linear fluorescence for optimal resolution for DNA, and data collected for 50,000 events per sample. The percentages of G0, G1, S and G2 phases were manually determined using FlowJo 10 software.

### Apoptosis and cytotoxicity assays

Single-cell suspensions containing 1×10^5^ cells/sample, as well as apoptosis control treated with Staurosporine (1 µM, 1 h) and dead cell control (2% DMSO, 1 h) were stained using the Annexin V–APC Kit (Biolegend) according to the manufacturer's instructions. Cells were washed once with Annexin V Binding buffer, then resuspended in 100 µl of the same buffer and stained using 5 µl Annexin V reagent. Samples were incubated for 15 min in the dark and then diluted with 400 µl binding buffer and adjusted to 50 mg/ml propidium iodide, prior to flow analysis on a Fortessa cytometer (Becton Dickinson: 640 nm laser, emission 670 nm). Data analysis was carried out using FlowJo 10 software.

For assessing Apoptosis/Cytotoxicity/Viability we used the ApoTox-Glow Triplex Assay (Promega G6320) according to the manufacturer's instructions. Cells were plated at 40,000 cells in 100 µl medium, per well of tissue culture 96-well plates and grown for a further 2 days. The viability/cytotoxicity reagent (20 µl) was added to each well, mixed by orbital shaking and incubated for 30 min at 37°C and then read at 405 nm (excitation)/505 nm (emission) for viability, and 485 nm (excitation)/520 nm (emission) using a BioTek Synergy Ht plate reader. To measure apoptosis, Caspase-Glo reagent (100 ml) was added to each well, mixed by orbital shaking, incubated at room temperature for 30 min and the luminescence was read using the Biotek Synergy Ht plate reader. Cells treated with ethanol (0.7%, 1 h) and staurosporine (1 mM, 1 h) served as cell death and apoptosis controls, respectively.

### Northern analysis of RNA

RNA was prepared from cells lysed in acid guanidinium hydrochloride (Chomczynski and Sacchi, 1987). Northern blots were produced from samples containing 10 µg of total RNA probed with the ^32^P-labelled Gab1α cDNA.

### Quantitative reverse transcriptase PCR

RNA (1 µg) purified using RNeasy Mini Kit (Qiagen) was used to sythesise cDNA using SuperScript First-Strand Synthesis System (Invitrogen). Approximately 1/60th of the cDNA was amplified using Platinum SYBR Green QPCR kit (Invitrogen) using the conditions; 50°C for 2 min, 95°C for 2 min followed by 40 cycles of 95°C for 15 s, 60°C for 30 s, with a final cycle consisting of 95°C for 1 min, 60°C for 30 s and 95°C for 15 s. Gab1 isoform-specific forward primers were: α, 5′-GGAGAAGAAGTTGAAGCGTTA-3′ and β, 5′-GACGCATGACACCTGAGTA-3′. The common reverse primer was: 5′-GCAACACAAACCACCTTCT-3′. β-actin control primers were: forward 5′-TGACAGGATGCAGAAGGAGA-3′, reverse 5′-GTACTTGCGCTCAGGAGGAG-3′.

### Immunoprecipitation and immunoblotting

Immunoprecipitations were performed essentially as described previously (Burdon et al., 1999). ESC (5×10^6^) were plated overnight in 10-cm-diameter dishes. The cells were serum and cytokine starved for 24 h prior to induction with growth factors. Cells were lysed in 0.6 ml ice-cold lysis buffer (150 mM NaCl, 10 mM Tris-HCl, pH 7.4, 0.5% NP40, 1 mM NaVO_4_, 1 mM EDTA, 0.5 mM PMSF), cleared of nuclear and cytoplasmic debris and then incubated with ∼1 µg antibody and Protein-A Sepharose for 4-16 h at 4°C. Immune complexes were washed extensively and then boiled in SDS sample buffer prior to gel electrophoresis. For protein analysis in whole cell lysates, ESCs were plated at 5×10^6^ cells per well in 6-well dishes. After plating overnight and incubation for a further 24 h in serum-free medium, cells were stimulated with growth factors, washed once with PBS and lysed in 100 or 200 µl of SDS sample buffer. These lysates were sonicated and boiled prior to loading on gels. Following electrophoresis on 8 or 10% denaturing SDS-polyacrylamide gels, proteins were electroblotted onto ECL-nitrocellulose filters (Amersham) and probed with antibodies (diluted 1:1000). The antibodies used in this study were obtained from New England Biolabs/Cell Signalling Technology: phospho-Erk, #9101; phospho-Akt (Ser473), #4058, phospho Gab1 Tyr307, #3234; phospho Gab1 Tyr627, #3231; phospho Stat3, #9131, #9138; from BD/Transduction Labs: Erk2 ,E16220; Stat3, S21320; Grb2, G16720; from Upstate Biotechnology: phosphotyrosine, 4G10; Gab1 CT, #06-579; p85, #06-195; and from Santa Cruz: SHP2, sc-280; Myc, sc-40; Oct4, sc-5279. A rabbit polyclonal anti-Gab1 antibody was very generously provided by T. Hirano and M. Hibi (Osaka University, Osaka, Japan) (Takahashi-Tezuka et al., 1998).

### Immunocytochemistry and confocal microscopy

Approximately 1×10^6^ cells in 500 µl of medium were allowed to attach to glass coverslips (pretreated with 1% gelatin) in the bottom of a 6-well culture dish for 1 h. Wells were then flooded with medium and the cells cultured overnight. Cells on the cover slips were fixed for 10 min in 4% paraformaldehyde at room temperature, gently washed five times with PBST (PBS, 0.03% Triton X-100), and then incubated with blocking solution (PBST, 3% goat serum, 1% BSA) for 1 h at room temperature. Primary antibodies were diluted in blocking solution and applied overnight at 4°C, followed by four washes with PBST. Appropriate secondary antibodies were diluted 1:1000 in blocking solution and applied for 1 h, at room temperature in the dark. The cells were washed extensively with PBST, and the final wash contained 10 µg/ml DAPI. The antibodies used were Oct4 primary antibody at 1:200 (Santa Cruz, sc5279) with goat-anti-mouse IgG2b secondary antibody, rabbit anti-Gab1 antibody at 1:500 (Takahashi-Tezuka et al., 1998). The coverslips were then laid over the depression in a concave microscope slide filled with 150 µl PBS and imaged using a Nikon EC1 confocal microscope at 60× magnification. For imaging live cells on microscope slides, cells were maintained in Opti-MEM (Life Technologies).

### ^3^H palmitate labelling

Transfected cells were incubated for 4 h in medium containing 0.5mCi/ml [^3^H]palmitic acid (Perkin Elmer) and 0.1% BSA (fatty acid free). The GFP-tagged proteins were then immunoprecipitated with magnetic microbeads coupled to GFP antibody (Miltenyi Biotech). Immunoprecipitated proteins were separated by SDS-PAGE and transferred to nitrocellulose membranes. [^3^H]palmitate present on the recovered GFP-tagged proteins was detected using a Kodak Biomax Transcreen LE intensifier screen.

### Statistical methods

Cell growth data were analysed using mixed models to allow for random variability in the group effects occurring between the replicate experiments. The models fitted: group, day and the group×day interaction as fixed effects; and replicate, replicate×group, replicate×day and replicate×group.day as random effects. A different residual variance was allowed for each day of the trial, after checking that this led to a significant improvement in the model compared with a model using a constant residual variation, using a likelihood ratio test. In order to satisfy normality assumptions, a log transformation of the data was used for all analyses except that for Fig. 3B where a square root transformation was preferable. Student's *t*-tests were defined with the models to carry out pairwise comparisons of specified groups on each day of the trial.

## Supplementary Material

Supplementary information
