# Supplementary material for: LIF-dependent survival of embryonic stem cells is regulated by a novel palmitoylated Gab1 signalling protein
Source: J Cell Sci. 2018 Sep 20;131(18):jcs222257. doi: 10.1242/jcs.222257 (PMC6176924; doi:10.1242/jcs.222257)
Supplement: Supplementary information [file joces-131-222257-s1.pdf]

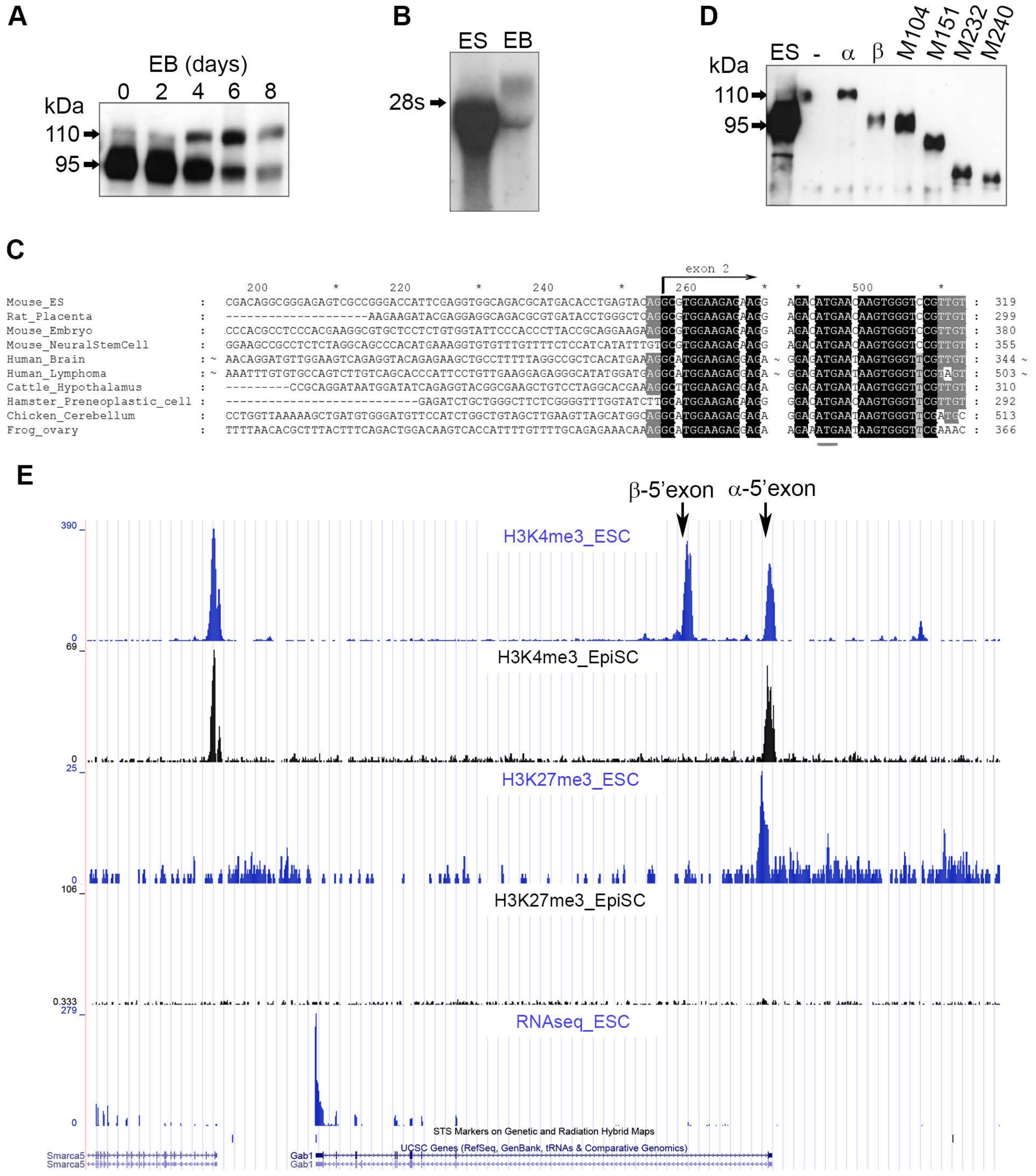
A
EB (days)
0 2 4 6 8
kDa
110
95
B
ES EB
28s
D
ES - α β M104 M151 M232 M240
kDa
110
95
C
exon 2
Mouse_ES
Rat_Placenta
Mouse_Embryo
Mouse_NeuralStemCell
Human_Brain
Human_Lymphoma
Cattle_Hypothalamus
Hamster_Preneoplastic_cell
Chicken_Cerebellum
Frog_ovary
E
β-5'exon α-5'exon
H3K4me3_ESC
H3K4me3_EpiSC
H3K27me3_ESC
H3K27me3_EpiSC
RNAseq_ESC
STS Markers on Genetic and Radiation Hybrid Maps
UCSC Genes (RefSeq, GenBank, tRNAs & Comparative Genomics)
Smarca5
Gab1

SUPPLEMENTARY FIGURE 1. **Expression of a novel form of Gab1.** (A) Western blot showing Gab1 protein expression in whole cell lysates prepared from undifferentiated ESCs, ESCs at 2, 4, 6 and 8 days during embryoid body differentiation in suspension culture. (B) Northern blot of total RNA prepared from ESC and day 8 embryoid body hybridised with Gab1 cDNA probe. The position of the 28S ribosomal RNA (approx. 5 kb) identified by ethidium bromide staining of the filter prior to hybridisation is indicated (C) Alignment of Gab1 ESTs which have a non-coding 5' exon analogous to Gab1β that have been identified in different species. (D) Gab1 proteins produced by COS7 cells transfected with Gab1α and β cDNAs where translation initiates at methionines 104, 151, 232 or 240. (E) H3K4me3 and H3K27me3 histone modifications at the mouse *Gab1* locus in ESC and EpiSCs visualised in the UCSC genome browser. Whereas a region adjacent to the Gab1β exon is enriched for the H3K4me3 mark indicative of active transcription, the corresponding region at the Gab1α exon is enriched for both active and repressed (H3K27me3) modifications (bivalent). The RNA-seq track in ESC demonstrates that the Gab1 locus is transcriptionally active (from right to left).

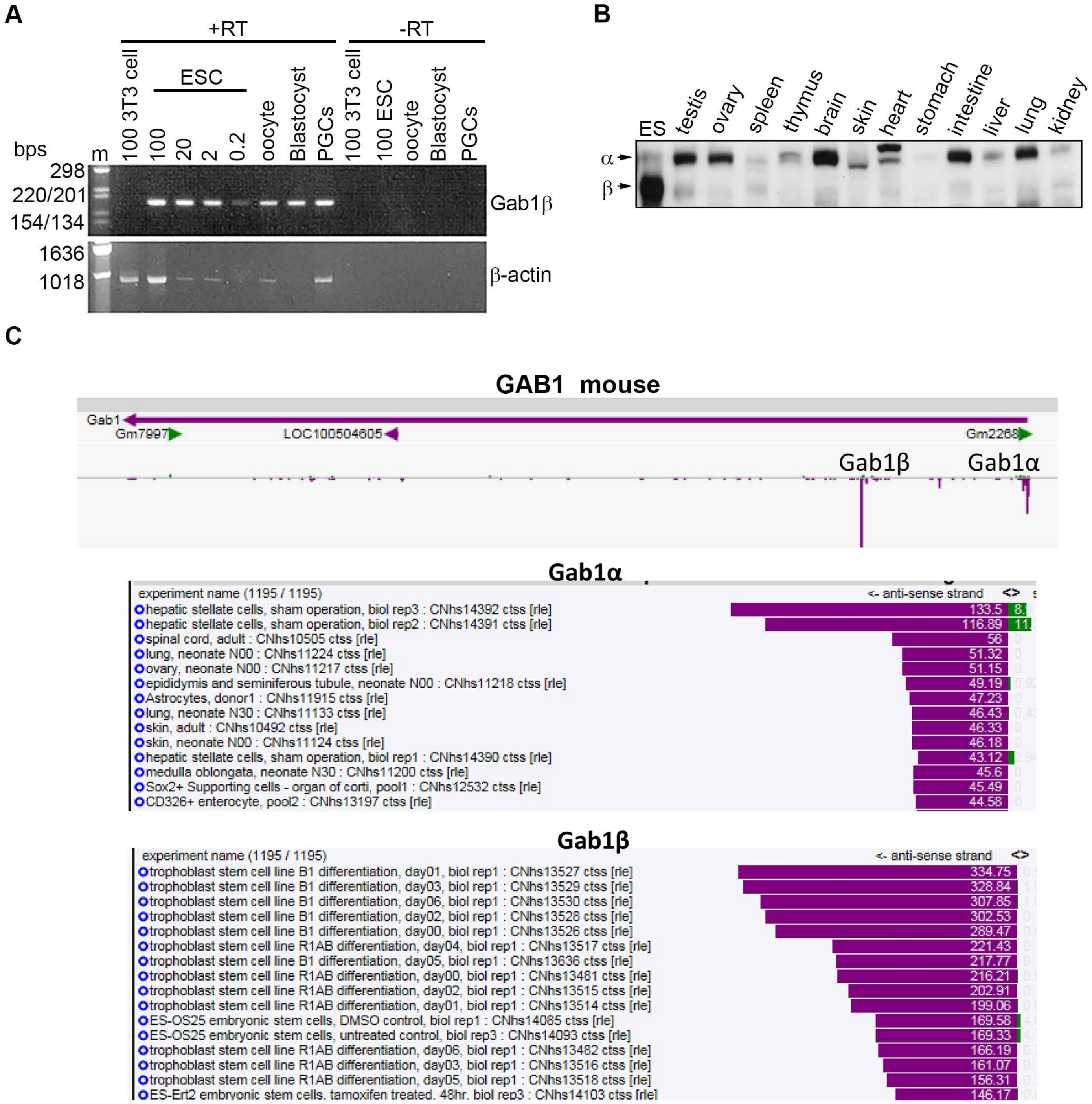
A
+RT
-RT
ESC
100 3T3 cell
100
20
2
0.2
oocyte
Blastocyst
PGCs
100 3T3 cell
100 ESC
oocyte
Blastocyst
PGCs
bps
m
298
220/201
154/134
1636
1018
Gab1β
β-actin
B
ES
testis
ovary
spleen
thymus
brain
skin
heart
stomach
intestine
liver
lung
kidney
α
β
C
GAB1 mouse
Gab1
Gm7997
LOC100504605
Gm2268
Gab1β
Gab1α
Gab1α
experiment name (1195 / 1195)
<- anti-sense strand
hepatic stellate cells, sham operation, biol rep3 : CNhs14392 ctss [rle] 133.5
hepatic stellate cells, sham operation, biol rep2 : CNhs14391 ctss [rle] 116.89
spinal cord, adult : CNhs10505 ctss [rle] 56
lung, neonate N00 : CNhs11224 ctss [rle] 51.32
ovary, neonate N00 : CNhs11217 ctss [rle] 51.15
epididymis and seminiferous tubule, neonate N00 : CNhs11218 ctss [rle] 49.19
Astrocytes, donor1 : CNhs11915 ctss [rle] 47.23
lung, neonate N30 : CNhs11133 ctss [rle] 46.43
skin, adult : CNhs10492 ctss [rle] 46.33
skin, neonate N00 : CNhs11124 ctss [rle] 46.18
hepatic stellate cells, sham operation, biol rep1 : CNhs14390 ctss [rle] 43.12
medulla oblongata, neonate N30 : CNhs11200 ctss [rle] 45.6
Sox2+ Supporting cells - organ of corti, pool1 : CNhs12532 ctss [rle] 45.49
CD326+ enterocyte, pool2 : CNhs13197 ctss [rle] 44.58
Gab1β
experiment name (1195 / 1195)
<- anti-sense strand
trophoblast stem cell line B1 differentiation, day01, biol rep1 : CNhs13527 ctss [rle] 334.75
trophoblast stem cell line B1 differentiation, day03, biol rep1 : CNhs13529 ctss [rle] 328.84
trophoblast stem cell line B1 differentiation, day06, biol rep1 : CNhs13530 ctss [rle] 307.85
trophoblast stem cell line B1 differentiation, day02, biol rep1 : CNhs13528 ctss [rle] 302.53
trophoblast stem cell line B1 differentiation, day00, biol rep1 : CNhs13526 ctss [rle] 289.47
trophoblast stem cell line R1AB differentiation, day04, biol rep1 : CNhs13517 ctss [rle] 221.43
trophoblast stem cell line B1 differentiation, day05, biol rep1 : CNhs13636 ctss [rle] 217.77
trophoblast stem cell line R1AB differentiation, day00, biol rep1 : CNhs13481 ctss [rle] 216.21
trophoblast stem cell line R1AB differentiation, day02, biol rep1 : CNhs13515 ctss [rle] 202.91
trophoblast stem cell line R1AB differentiation, day01, biol rep1 : CNhs13514 ctss [rle] 199.06
ES-OS25 embryonic stem cells, DMSO control, biol rep1 : CNhs14085 ctss [rle] 169.58
ES-OS25 embryonic stem cells, untreated control, biol rep3 : CNhs14093 ctss [rle] 169.33
trophoblast stem cell line R1AB differentiation, day06, biol rep1 : CNhs13482 ctss [rle] 166.19
trophoblast stem cell line R1AB differentiation, day03, biol rep1 : CNhs13516 ctss [rle] 161.07
trophoblast stem cell line R1AB differentiation, day05, biol rep1 : CNhs13518 ctss [rle] 156.31
ES-Ert2 embryonic stem cells, tamoxifen treated, 48hr, biol rep3 : CNhs14103 ctss [rle] 146.17

SUPPLEMENTARY FIGURE 2. **Gab1β expression profiles.** (A) Reverse transcriptase PCR analysis of Gab1β expression in embryonic cells. Gab1β expression was analysed in cDNA prepared from ESC (0.2-100 ESC equivalents), unfertilised oocytes, blastocysts and primordial germ cells (PGCs) and NIH3T3 fibroblasts (equivalent to 100 cells). Gab1β, and β-actin specific primers generated 184 bp and 939 bp amplicons, respectively. (B) Western blot of Gab1 protein expression in whole tissue lysates prepared from adult mouse. (C) Quantitative transcription start site expression at the mouse *Gab1* gene locus derived from FANTOM5 mouse Cap Analysis of Gene Expression (CAGE) data analysis. The upper sections describes the summary of transcription initiation at Gab1α and Gab1β transcription start sites (TSS) combining cumulative CAGE data from over 1000 individual mouse cell and tissue samples visualised in the ZENBU genome browser. The tissues/cell types with the highest expression (in descending order) for Gab1α and Gab1β TSS is presented as normalised tags per million counts (purple bars on right) for each sample.

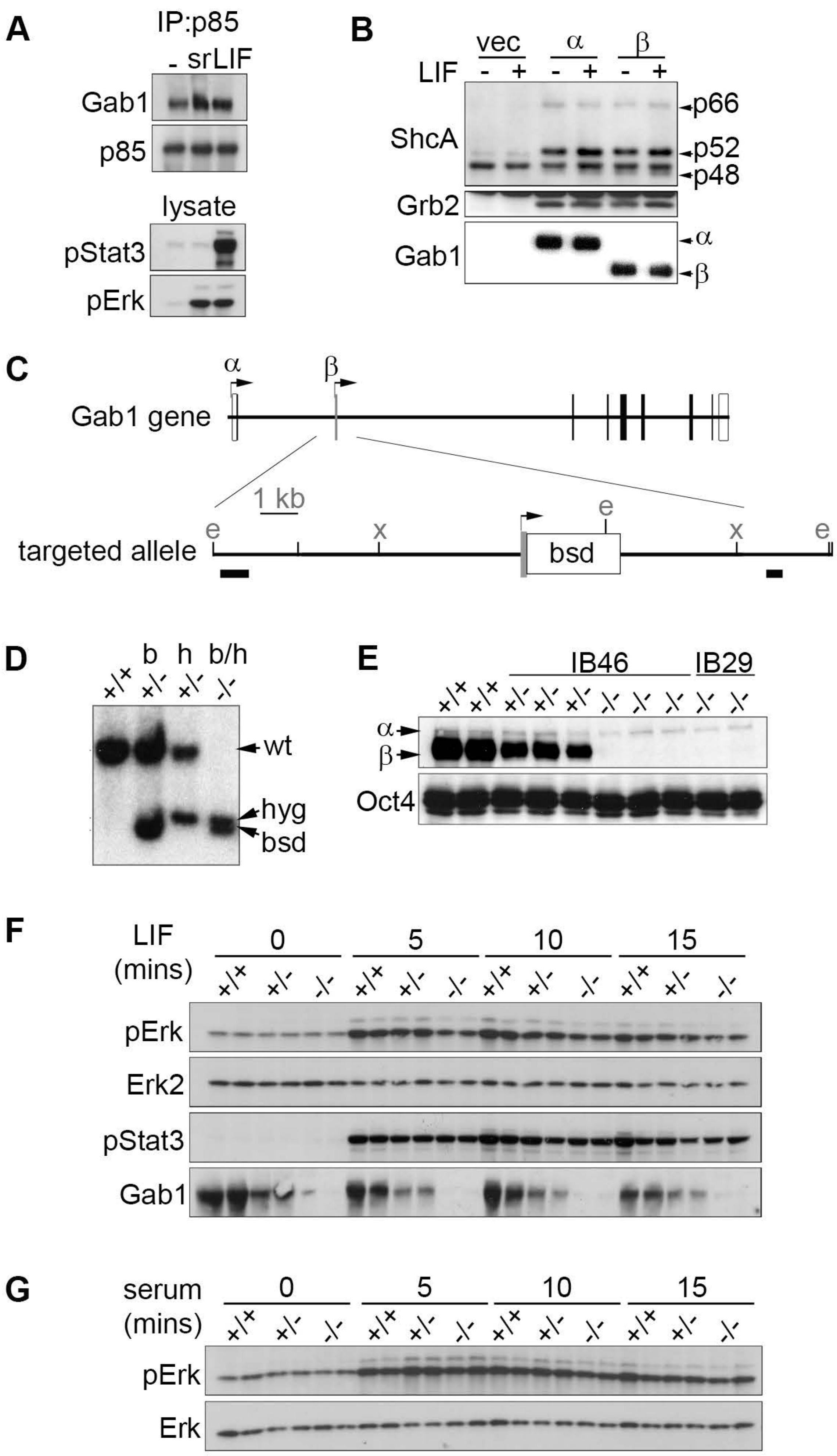

Journal of Cell Science • Supplementary information

SUPPLEMENTARY FIGURE 3. **Analysis of Gab1β signalling** (A) Western blots of PI3K p85 immunoprecipitates from unstimulated ESC (-) or ESCs stimulated with serum (sr) or LIF (lf) for 10 minutes, incubated with Gab1 and p85 antibodies; and cell lysates generated in (A) incubated with antibodies against phospho-Stat3 (Y705) and phospho-Erk. (B) Western blot of anti-myc immunoprecipitates prepared from ESCs transfected with empty (vec), myc epitope-tagged Gab1α and myc epitope-tagged Gab1β expression vectors, incubated with antibodies against Shc, Grb2 and Gab1. (C) Schematic showing the Gab1 gene locus and targeting vector used to insert the blastocydin^R selection cassette into the Gab1β first exon. Location of external probes used to screen for targeted insertion of the selection cassette are shown as black bars. (D) Southern blot of EcoRV digested genomic DNA prepared from IOUD2 Gab1β wild-type ESC (+/+), heterozygous Gab1β-blastocidin^R, Gab1β-hygromycin^R clones (+/-) and a double targeted Gab1β-blastocidin^R/Gab1β-hygromycin^R homozygous Gab1β null clone (-/-), hybridised with a ^{32}P-labelled Gab1 5' external probe. (E) Western blot of Gab1 and Oct4 protein expression in whole cell lysates from Gab1β wildtype, Gab1β heterozygous and Gab1β null ESCs. (F, G) Western blot analysis of phospho-Erk, Erk2 and phospho-Stat3 (Y705) in cell lysates prepared from Gab1β wild-type, heterozygous and homozygous null ESCs following stimulation with LIF or Serum for 0,5,10 and 15 minutes.

Journal of Cell Science • Supplementary information

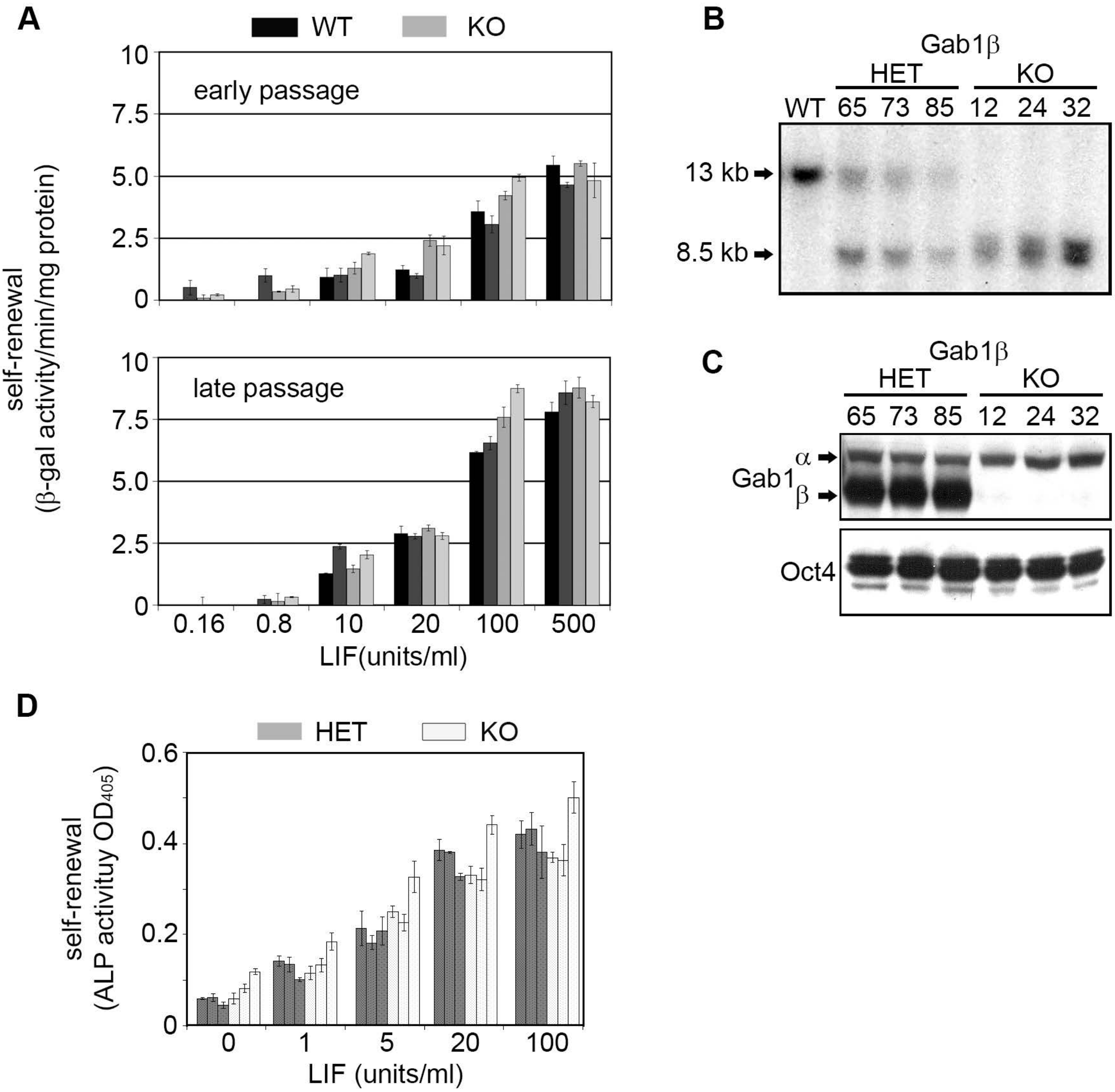

Journal of Cell Science • Supplementary information

SUPPLEMENTARY FIGURE 4. **Role of Gab1β in ESC self-renewal.** (A) Self-renewal response to LIF titration in two Gab1β wild-type (darker shaded bars) and Gab1β KO (light shaded grey bars) IOUD2 cell lines as measured by *pou5F1*-LacZ activity in day 5 low density cultures at early and late passages. Data represents the means ± SD of three biological replicates. (B) Southern blot of EcoRV digested genomic DNA prepared from E14Tg2a wild-type ESC (WT), three heterozygous Gab1β-hygromycin^{R} clones (HET) and three Gab1β-blastocidin^{R}/Gab1β-hygromycin^{R} homozygous Gab1β null clones (KO) , hybridised with a ^{32}P-labelled Gab1 5' external probe. (C) Western blot of Gab1 and Oct4 protein expression in cell lysates prepared from E14Tg2a Gab1β HET and KO clones. (D) Self-renewal response to LIF titration in Gab1β HET (dark bars) and Gab1β KO (white bars) E14Tg2a ESCs as measured by alkaline phosphatase activity. Data represents the means ± SD of three biological replicates.

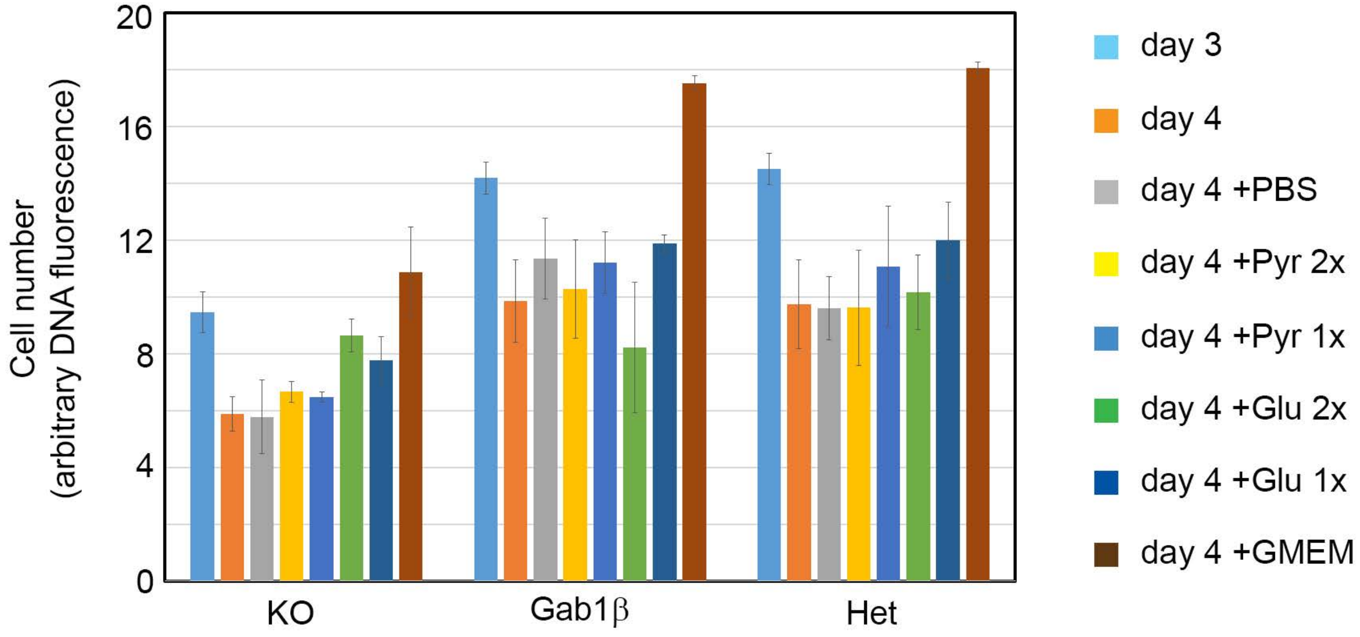

SUPPLEMENTARY FIGURE 5. **Assessment of the factors that may limit ESC growth in near-confluent cultures.** (A) Growth of Gab1β KO, Gab1β expressing, and Gab1β Heterozygous ESCs at 3 days in culture, and then after supplementation with 10 μl PBS or PBS containing 1 mM or 2 mM Sodium Pyruvate, or 2 mM or 4 mM Glutamine, or 10 μl GMEM and cultured for a further 24 hrs.

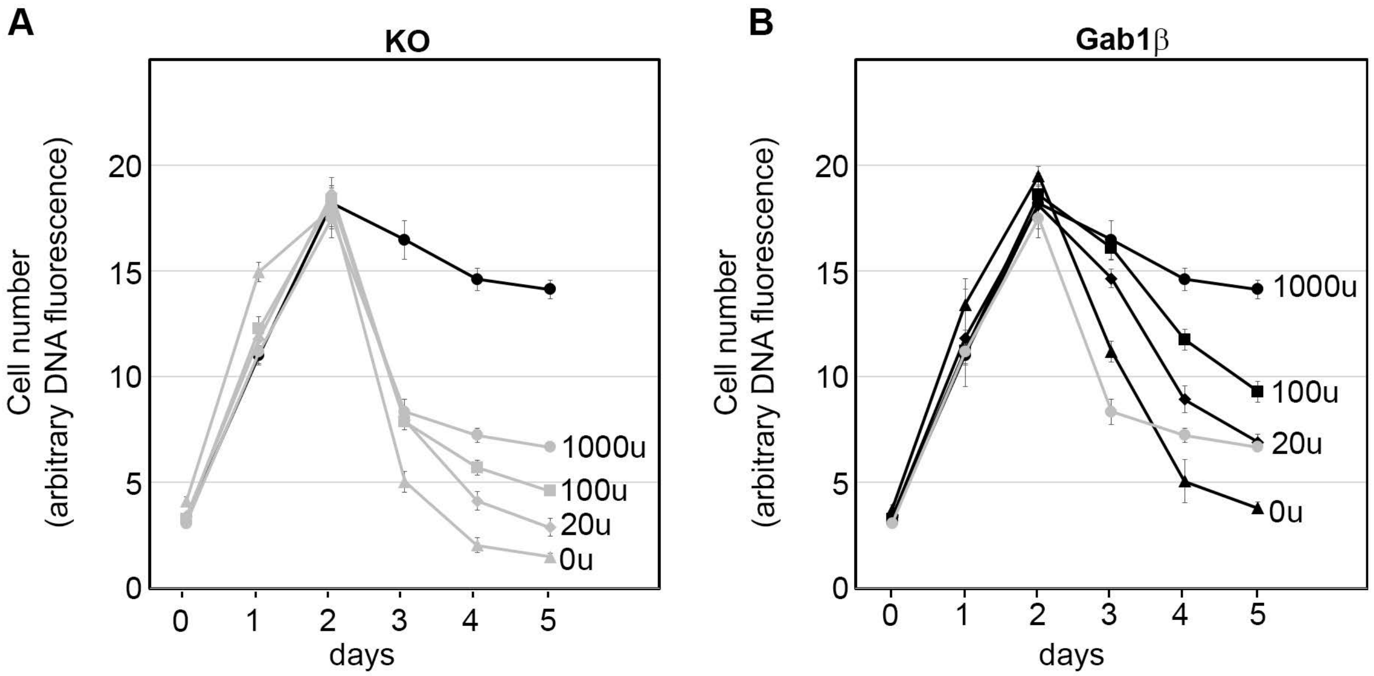

SUPPLEMENTARY FIGURE 6. **Effect of LIF titration on growth of Gab1β KO and Gab1β expressing ESCs.** Growth profiles of (A) Gab1β KO and (B) Gab1β expressing ESCs cultured in the presence 0, 20, 100 and 1000 units/ml of LIF for 6 days. The values represent the means of four biological replicate cultures +/- SD. For reference, the dark line in (A) is comprised of the values obtained from the Gab1β expressing ESCs grown in 1000 units/ml LIF , and in (B) the grey line is the KO cells grown in 1000 units/ml LIF.
